# Kazald1 attenuates chondrocyte fibrosis to potentiate hyaline cartilage regeneration by interfering with the pro-fibrotic TGF-β signaling

**DOI:** 10.7150/thno.113604

**Published:** 2025-09-08

**Authors:** Yue Zhu, Haoyang Liu, Yuting Zhang, Danning Zheng, Yaning Li, Jialin Chen, Jiake Xu, Wei Zhang

**Affiliations:** 1 School of Medicine, Jiangsu Key Laboratory for Biomaterials and Devices, Southeast University, Nanjing, 210000, China.; 2 Shenzhen University of Advanced Technology, and Shenzhen Institutes of Advanced Technology, Chinese Academy of Sciences, Shenzhen, 518055, China.; 3 Department of Ophthalmology, Zhongda Hospital, Southeast University, 210009, Nanjing, China.; 4 China Orthopedic Regenerative Medicine Group (CORMed), 310058, Hangzhou, China.; 5 School of Biomedical Sciences, The University of Western Australia, Perth, Western Australia, 6000, Australia.

**Keywords:** cartilage regeneration, Kazald1, TGF-β, chondrocyte fibrosis, fibrocartilage

## Abstract

**Background:** Cartilage regeneration remained a significant challenge, often leading to the formation of mechanically inferior fibrocartilage instead of physiological hyaline cartilage. Currently, there were no effective treatments for cartilage fibrosis, necessitating the exploration of potential molecular targets.

**Methods:** We perform single-cell sequencing of rat spontaneously formed fibrocartilage following osteochondral injury and rat normal hyaline cartilage with a comprehensive analysis of the heterogeneous cell subpopulations between two groups. Subsequently, we express and purify the full length of recombinant human Kazald1 protein (aa 31-304) with predicted tertiary and secondary structures, and determine its anti-fibrotic effect and explore its regulatory mechanisms using in vitro cultured chondrocytes, in the presence or absence of the pro-fibrotic factor TGF-β1. Finally, we evaluate the therapeutic potential of recombinant Kazald1 protein in promoting hyaline cartilage regeneration and maintenance using rat osteochondral injury models and human cartilage explants, respectively.

**Results:** Through single cell sequencing of hyaline cartilage and fibrocartilage, we identified Kazald1 as a key molecule in maintaining cartilage homeostasis. During cartilage fibrosis, Kazald1 expression was significantly down-regulated and becomes imbalanced with TGF-β1. Recombinant Kazald1 protein effectively inhibited TGF-β1-induced chondrocyte fibrosis and preserves chondrocyte phenotype. Mechanistically, Kazald1 formed a dimer with TGFBR1, blocking the pro-fibrotic TGF-β1-Akt/Smad3 signaling and suppressing the expression of fibrotic genes. In rat models of cartilage injuries, the combination of Kazald1 and TGF-β1 effectively promoted hyaline cartilage regeneration with structural restoration and functional recovery. This combination also enhanced hyaline cartilage maintenance and inhibited TGF-β1-induced cartilage fibrosis in human cartilage explants.

**Conclusion:** This study unveils the pivotal role of Kazald1 in the regulation of cartilage fibrosis and highlights its potential as a therapeutic agent for facilitating hyaline cartilage regeneration.

## Introduction

Articular cartilage, the connective tissue that covers joint surfaces, is a smooth, white hyaline cartilage essential for reducing friction and distributing loads during movement. Derived from the mesoderm, articular cartilage lacks innervation and vascularization 1, 2, with chondrocytes being the sole cell type present, surrounded by a dense extracellular matrix (ECM) principally composed of type II collagen (COL2) and proteoglycans 3. Articular cartilage injuries are common joint issues, especially among physically active individuals. The treatment of articular cartilage injuries presents a significant challenge, not only due to the cartilage's limited spontaneous healing capacity but also because such injuries often lead to post-traumatic osteoarthritis (OA) 4, 5. Various reparative and restorative techniques, such as bone marrow stimulation and more advanced autologous chondrocyte implantation (ACI), are commonly employed to treat cartilage injuries in clinical practice 6, 7. Although these methods can lead to the formation of a rudimentary repair tissue, it is often mechanically inferior fibrocartilage expressing type I collagen (COL1), rather than the more robust hyaline cartilage containing COL2 8. This aberrant fibrocartilage lacks the mechanical properties necessary to withstand repetitive loading, potentially leading to cartilage degeneration and OA progression 3. This fibrotic transition is also commonly observed during the *in vitro* monolayer culture of chondrocytes, a critical step in ACI. During this process, chondrocytes undergo morphological changes from a rounded to a fibroblastic shape and exhibit molecular changes that result in the production of COL1 instead of COL2, a phenomenon known as chondrocyte dedifferentiation. Restoring chondrocytes to their healthy phenotype and regenerating articular cartilage to its natural hyaline cartilage state, rather than merely repairing it with fibrocartilage, is the central challenge behind the intensive research in cartilage regenerative medicine 3.

Various biological molecules, including growth factors, small molecules, cytokines and peptides, have been investigated for their roles in regulating cartilage homeostasis and improving cartilage regeneration 9, 10. Among these, factors from the transforming growth factor-beta (TGF-β) family are the most significant and have been extensively researched in cartilage regenerative medicine. TGF-β plays an important role in cartilage development, regulating key phases of chondrogenesis, including mesenchymal condensation, chondroprogenitor proliferation, ECM deposition and terminal differentiation 11. In arthrofibrosis, TGF-β is involved in the signaling pathways of cytokines and mediators that drive fibroblasts to differentiate into myofibroblasts 12. In mature articular cartilage, TGF-β is highly expressed in healthy cartilage to maintain chondrocyte homeostasis. However, the expression of TGF-β is significantly down-regulated following cartilage injury or disease 13, 14. Due to the critical role of TGF-β in cartilage development and maintenance, numerous studies have explored the use of exogenous TGF-β to promote chondrogenesis and treat cartilage injuries in cartilage tissue engineering and regenerative medicine. *In vitro* supplementation of TGF-β can promote chondrogenesis of stem cells (thus TGF-β is considered a necessary chondrogenic induction factor in chondrogenic medium) and stimulate chondrocytes proliferation and ECM formation 15, but it often leads to a fibrotic and hypertrophic phenotype 16, 17. Furthermore, *in vivo* administration of exogenous TGF-β following cartilage injury can promote the chondrogenic differentiation of mesenchymal stem cells (MSCs), enhance chondrocyte proliferation and migration, and ultimately facilitate the healing of cartilage defects 18, 19. However, such administration often results in undesired chondrocyte fibrosis and fibrocartilage formation 20, 21. Indeed, TGF-β signaling is widely recognized as a classic pathway that induces fibrosis in multiple organs, including the kidneys, heart, and liver 22. Interestingly, while TGF-β is highly expressed during cartilage development and in normal hyaline cartilage, it does not cause cartilage fibrosis in a healthy state. This suggests the involvement of specific regulatory molecules that counteract the pro-fibrotic effects of TGF-β, thereby allowing TGF-β to exert a stable chondrogenic effect and maintain cartilage homeostasis by reducing chondrocyte fibrosis. Identifying and understanding these regulatory mechanisms is essential for boosting the therapeutic efficacy of TGF-β in treating cartilage injuries and advancing cartilage regenerative medicine.

To elucidate the molecular mechanisms involved in cartilage homeostasis maintenance, and identify potential molecular targets for hyaline cartilage regeneration, in the present study, we employed single-cell RNA sequencing (scRNA-seq) to investigate the transcriptional differences between hyaline cartilage and fibrocartilage at a high-throughput single-cell level. Through analysis of 25,789 cells from rat hyaline cartilage and fibrocartilage, we identify a candidate Kazal Type Serine Peptidase Inhibitor Domain 1 (Kazald1), which is significantly down-regulated in the spontaneously formed fibrocartilage following cartilage injuries. Kazald1, also known as IGFBP-rP10, BONO1, FKSG28, or FKSG40 23, encodes a secreted member of the IGFBP superfamily. To date, only a few publications related to Kazald1 are documented in the PubMed database. These studies primarily focus on cancer, limb, and bone research. In tumorigenesis, Kazald1 methylation level is highly correlated with glioma progression 23. During limb regeneration in salamanders, Kazald1 expression is undetectable before intact limb amputation but increases shortly after amputation, is maintained during the embryonic stage of regeneration, and drastically down-regulates at the end of regeneration 24. Kazald1-morpholino-treated animals exhibit delayed chondrification and differentiation of the digits at the palette-to-early-digit stage, indicating a critical role for Kazald1 in regulating limb regeneration 24. Additionally, Kazald1 is expressed in osteoblasts during bone regeneration and promotes the proliferation of osteoblastic cells *in vitro* 25. However, whether Kazald1 plays a significant role in regulating TGF-β-induced chondrocyte fibrosis and maintaining cartilage homeostasis, or whether Kazald1 can be utilized therapeutically for enhanced hyaline cartilage regeneration, remains unknown.

Here, by combining scRNA-seq, recombinant protein production, and experimental validation at the cellular, animal, and human levels, we investigate whether Kazald1 acts as a key regulator in TGF-β1-induced chondrocyte fibrosis and fibrocartilage formation, as well as explore its therapeutic potential for hyaline cartilage regeneration. Our results indicate that Kazald1 attenuates TGF-β1-induced chondrocyte fibrosis by interfering with the pro-fibrotic TGF-β1 signaling through binding to TGF-β1 receptor. This interaction suppresses the activation of downstream Akt/Smad3, thereby inhibiting the expression of fibrotic genes to preserve chondrocyte phenotype. A combination treatment of Kazald1 and TGF-β1, delivered within a biocompatible hydrogel carrier, effectively promotes hyaline cartilage regeneration with structural restoration and functional recovery in rat cartilage injury models. Furthermore, we confirm that the Kazald1 and TGF-β1 combination improves hyaline cartilage maintenance in human cartilage explants.

## Materials and Methods

### Sprague-Dawley (SD) rat osteochondral injury model

The animal study protocol was approved by the Ethics Committee of Southeast University (20221010010). All experimental procedures were carried out by following the approved guidelines. SD rats (200 ± 20 g) were anesthetized with sodium pentobarbital by intraperitoneal injection at a dosage of 50 mg/kg. Subsequently, a cylindrical osteochondral defect (2 mm in height, 2 mm in diameter) was created on the right patellar groove, while the left side remained uninjured, according to the assigned group. After surgery, the rats were allowed to move freely, and were provided with standard food and water. All rats were sacrificed at 4 weeks post-surgery, and samples of the left normal cartilage and right repaired cartilage were collected for histological staining and scRNA-seq analysis.

### scRNA-seq

Normal cartilage samples (n = 8) and repaired fibrocartilage samples (n = 8) were thoroughly washed three times with Hanks' balanced salt solution (HBSS). The sequencing read depth was >30,000 reads per cell in all samples. Single-cell suspensions (2×10^5^ cells/ml) in phosphate-buffered saline (PBS, HyClone) were loaded onto microwell chip using the Singleron Matrix® Single Cell Processing System. Barcoding beads were subsequently collected from the microwell chip, and the captured mRNA was reverse transcribed into cDNA. The resulting cDNA was then amplified by PCR, fragmented, and ligated with sequencing adapters. The scRNA-seq libraries were constructed according to the protocol of the GEXSCOPE® Single Cell RNA Library Kits (Singleron) 26. Individual libraries were diluted to 4 nM, pooled, and sequenced on Illumina novaseq 6000 with 150 bp paired end reads. Raw reads were processed to generate gene expression profiles using CeleScope v1.12.0 (Singleron Biotechnologies) with default parameters. Scanpy v1.9.1 was used for quality control, dimensionality reduction and clustering under Python 3.7.

### Human Kazald1 protein expression and purification

To express recombinant Kazald1 protein, human Kazald1 cDNA (855 bp from NM_030929.5) encoding human Kazald1 (aa 31-304) was designed by codon-optimization and then cloned into the NdeI/XhoI sites of pET30a. The plasmid was named pET-30a-Kazald1 with Kanamycin (+) resistance and used to express human Kazald1 (aa 31-304) recombinant protein with an N terminal methionine and a C terminal polyhistidine-tagged (His-tagged). To express human Kazald1 (aa 31-304) recombinant proteins, bacteria, BL21 (DE3) transformed with pET-30a-Kazald1 were grown with induction of 0.2 mM IPTG at 15 ^o^C overnight. The recombinant proteins were detected as 30.9 kDa with an estimated isoelectric point of 4.86. The recombinant proteins were then purified using Nickle 2+ iminodiacetic acid groups (IDA) resin for affinity purification of His-tagged proteins (DGpeptides, Hangzhou, China). The purified His-tagged proteins were eluted with an elution buffer (0.5 M imidazole, 0.15 M NaCl with 50 mM sodium phosphate buffer pH 7.8). The eluted proteins were dialyzed with PBS, with three buffer exchanges. The purified proteins were estimated to be over 95% of purity on a sodium dodecyl sulfate-polyacrylamide gel electrophoresis (SDS-PAGE), along with bovine serum albumin (BSA).

### Cell culture

Human chondrocyte (C28/I2) and mouse chondrocyte (ATDC5) cell lines were purchased from Shanghai Cell Bank (Shanghai, China). Growth medium containing DMEM high-glucose (H-DMEM, Gibco, Carlsbad, CA) with 10% fetal bovine serum (FBS, Wisent, Canada) and 1% penicillin-streptomycin (P/S, Gibco, Carlsbad, CA) was used for culture of both cell types. Cells were incubated in an incubator at 37 °C with 5% CO_2_, and the medium was changed every three days. If indicated, cells were treated with Kazald1 (10/50/100/200/500 ng/mL) and TGF-β1 (10 ng/mL, PeproTech, USA) for further experiment.

### Cell proliferation

Cell proliferation was measured by the CCK-8 kit (APExBIO, USA). Briefly, cells were cultured in 96-well plates for 1, 3, 5 days with a seeding density of 10^3^ cells/well. At the designed time point, the medium was replaced by a 10% CCK-8 working solution and was incubated in an incubator at 37 °C with 5% CO_2_ for 1 h. The absorbance of the incubated working solution was measured at 450 nm wavelength using a microplate reader (BioTek, USA).

### Safranin O staining

Chondrocytes were cultured in 96-well plates with a growth medium supplemented with Kazald1 and/or TGF-β1 for 3 and 7 days. Safranin O staining (Solarbio, China) was used to assess the production levels of cartilaginous glycosaminoglycans (GAGs). The staining intensity was calculated from the mean gray value (integrated density/area) quantified using ImageJ software (NIH, Bethesda, MD) and was then normalized to the control group.

### Total RNA isolation and quantitative real-time reverse transcriptase-polymerase chain reaction (qRT-PCR)

Total RNA extraction was performed using the RNA prep Pure Cell/Bacteria Kit (Tiangen, China) in accordance with the manufacturer's instructions. The RNA concentration was subsequently measured, and cDNA was synthesized via reverse transcription using the cDNA Reverse Transcription Kit (Toyobo, Japan). qRT-PCR was conducted with the SYBR Green qPCR Kit (Accurate Biotechnology Co., Ltd., China). The primer sequences for the target genes (GenScript, China) are shown in Table 1.

### Lentiviral constructs and chondrocyte infection

Lentiviral vectors for Kazald1 knockdown and overexpression were constructed by Obio Technology (Shanghai, China), wherein knockdown was mediated by three validated shRNAs (shKazald1-1: 5′-GATCGTGTCACATCCATATGA-3′; shKazald1-2: 5′-GCTAGCTTGACAGTGCTCACA-3′; shKazald1-3: 5′-GAGTGAAGAGAATGACGATTA-3′) cloned into pSLenti-U6-shRNA-CMV-EGFP-F2A-Puro-WPRE alongside a non-targeting scrambled shRNA control; meanwhile, overexpression leveraged the full-length human Kazald1 open reading frame fused to a C-terminal 6×His tag cloned into pSLenti-EF1-EGFP-F2A-Puro-CMV-MCS-WPRE. Human chondrocytes were seeded in 6-well plates at 5×10⁴ cells/well and grown to 60-70% confluency prior to lentiviral infection at a multiplicity of infection (MOI) of 90 in complete medium supplemented with 8 μg/mL polybrene. Following 24 h incubation, viral supernatants were replaced with fresh growth medium. Cells were subsequently stimulated with TGF-β1 (10 ng/mL, PeproTech, USA) for 48 h, after which total RNA was extracted for subsequent RT-qPCR analysis.

### Western blot

Total protein was extracted from chondrocytes using RIPA lysis buffer (Beyotime, China) supplemented with 1% Phenylmethanesulfonyl fluoride (PMSF, Beyotime, China). Protein concentration was determined using BCA protein assay kit (Beyotime, China). The extracted proteins were separated by gel electrophoresis and then blotted onto polyvinylidene difluoride membranes (Beyotime, China). The membranes were blocked with QuickBlock Blocking Buffer (Beyotime, China) and incubated with primary antibodies (Table S2) overnight at 4 °C.

Next, the membrane was washed with Tris Buffered Saline with Tween (TBST, Beyotime, China) and incubated with respective secondary antibodies (Table S2). The membranes were visualized using Western ECL Substrate (Beyotime, China), and the bands were imaged by an Imaging System (5200, Tanon, China). The densitometry value of the bands was determined using ImageJ software (NIH, Bethesda, MD).

### SD rat prophylactic treatment model of osteochondral injuries

SD rats (200 ± 20 g) were anesthetized via intraperitoneal injection of pentobarbital sodium at 50 mg/kg. A cylindrical osteochondral defect (2 mm in height, 2 mm in diameter) was created on the patellar groove, and 10 µL of Gel, Gel@K, Gel@T, or Gel@KT hydrogels were injected into the defect sites of the rats, according to the assigned group. Immediately following injection, UV irradiation (wavelength: 365-370 nm; light intensity: 50 mW cm^-2^) was applied for 20 s to induce gelation. All rats were sacrificed 4 weeks post-surgery, and the cartilage samples were collected for macroscopic, histological and functional evaluations.

### SD rat therapeutic treatment model of osteochondral injuries

SD rats (200 ± 20 g) were anesthetized via intraperitoneal injection of pentobarbital sodium at 50 mg/kg. Subsequently, a cylindrical osteochondral defect (2 mm in height and 2 mm in diameter) was created on the patellar groove. Four-weeks post-surgery, 10 µL of Gel, Gel@K, Gel@T, or Gel@KT hydrogels were injected into the defect sites, according to the assigned group. The gelation process was consistent with that used in the prophylactic treatment model. All rats were sacrificed 8 weeks post-surgery, and the cartilage samples were collected for macroscopic and histological evaluations.

### Immunofluorescent and immunohistochemical staining

Cell fixation was performed with 4% paraformaldehyde for 1 h. Tissue sections were subjected to antigen retrieval in citrate buffer (Beyotime, China) at 98 °C for 1 h. After blocking with QuickBlock Blocking Buffer (Beyotime, China), samples were incubated overnight at 4 °C with primary antibodies (Table S2), then with HRP-conjugated secondary antibodies (Beyotime, China) at room temperature for 2 h. CoraLite488-conjugated anti-rabbit secondary antibodies were used for sections for 1 h under a dark condition for immunofluorescent staining. Quantitative analysis was performed on randomly selected microscopic images from each group. The staining intensity was calculated from the mean gray value (integrated density/area) quantified using ImageJ software (NIH, Bethesda, MD) and normalized to the control group.

### Gait analysis

Gait analysis was performed with the CatWalk XT (Version 10.6) automated gait analysis system (Noldus, Netherlands) of rats at 1- , 2- and 4-week post-surgery according to a previous study 27. At each designated time point, each rat was allowed to walk freely on a glass walkway. Upon entering the region of interest, paw print recording was automatically initiated, with the built-in software classifying the prints from the right forelimb, left forelimb, right hindlimb, and left hindlimb. Only recordings with a stable walking speed (variation < 30%) were utilized for further analysis. The following parameters were assessed: max contact mean intensity, print area (cm^2^), max contact area (cm^2^), stands (s), and stride length (cm).

### Molecular docking

The crystal structure of TGFBR1 protein (Uniprot ID: P36897) and AlphaFold structure of Kazald1 protein (Uniprot ID: Q96I82) were downloaded from the RCSB Protein Data Bank (http://www.rcsb.org/). The optimized protein structures were carefully treated in several steps including residue repairing, protonation, and partial charges assignment in AMBER ff14SB force field. Protein-protein blind docking with TGFBR1 and Kazald1 was initiated using HDOCK server (https://hdock.phys.hust.edu.cn/). Top 10 predictions of complexes were obtained. Based on the scoring and binding modes, the top-ranked complex was selected for binding mode analysis. The results of molecular docking are visualized in PyMOL.

### Immunoprecipitation (IP)

HEK-293T cells were cultured to ~ 60% confluent in a 10 cm dish, and then transfected with TGFBR1 (NM_001130916.3) plasmid (pcDNA3.1-TGFBR1) by Lipofectamine® 2000 (Invitrogen, 11668-019). After 48 h, the cells were collected and lysed with IP lysis buffer. Each sample containing 10 μL of protein A/G magnetic beads (ThermoFisher Scientific, 88802) was incubated with 1μg recombinant Kazald1-Fc protein or 1 μg Fc fragment overnight at 4 °C. After washing three times with PBS containing 0.05% Tween-20, the cell lysates (containing -1 mg of total protein) were added to each sample and incubated for 1 h at room temperature with gentle rotation. The eluted immunocomplexes were then subjected to SDS-PAGE and immunoblotted with the anti-TGFBR1 antibody (abcam, ab235578) and the HRP-conjugated Mouse anti-Rabbit IgG Light Chain (Abclonal, AS061).

### Human cartilage explant culture

This protocol was approved by the Ethics Committee of Zhongda Hospital Affiliated to Southeast University (2022ZDSYLL415-P01) and Nanjing Drum Tower Hospital (2022-176-02). Human knee articular cartilage samples were obtained from OA patients, who underwent total knee arthroplasty, with informed consent obtained. Patient information is provided in Table S4. The cartilage tissue for explant culture was harvested from the region adjacent to the OA-affected area that exhibited no obvious signs of degeneration. Employing aseptic techniques, full-thickness cylindrical cartilage fragments were excised from the lateral and medial trochlear ridges of the distal femur using a biopsy punch. The articular cartilage explants were cultured in growth medium in an incubator at 37 °C with 5% CO_2_ for 3 days to maintain viability. Subsequently, Kazald1 and/or TGF-β1 were added, and the explants were further incubated for 7 days. The samples were then subjected to histological evaluation.

### Macroscopic and histological assessment

The repaired cartilage was photographed and evaluated using the International Cartilage Repair Society (ICRS) macroscopic assessment scale (Table S3) 28. Subsequently, cartilage samples were fixed in 4% paraformaldehyde and decalcified in 10% EDTA. After being embedded in paraffin and sectioned into 5 um slices, the sections were stained with toluidine blue (Solarbio, China), safranin O/fast green (Solarbio, China) and picrosirius red (Solarbio, China) to determine ECM production and organization. The staining intensity of toluidine blue and safranin O/fast green was quantified using ImageJ software (NIH, Bethesda, MD). Sections stained with picrosirius red were scanned under polarized light using the VS200 Research Slide Scanner (Olympus, Japan), and the organization of newly-formed collagen fibers was assessed using the Directionality plugin of ImageJ (NIH, Bethesda, MD).

### Statistics

Statistical analysis was conducted using GraphPad Prism Software. To compare two groups, an unpaired two-tailed Student's t-test was utilized. For comparisons involving three or more groups, one-way ANOVA followed by Tukey's multiple comparisons was applied. Data are presented as means ± standard deviation (SD). Differences are considered statistically significant when p < 0.05.

## Results

### Kazald1 is implicated in the regulation of cartilage fibrosis

**Figure 1:**
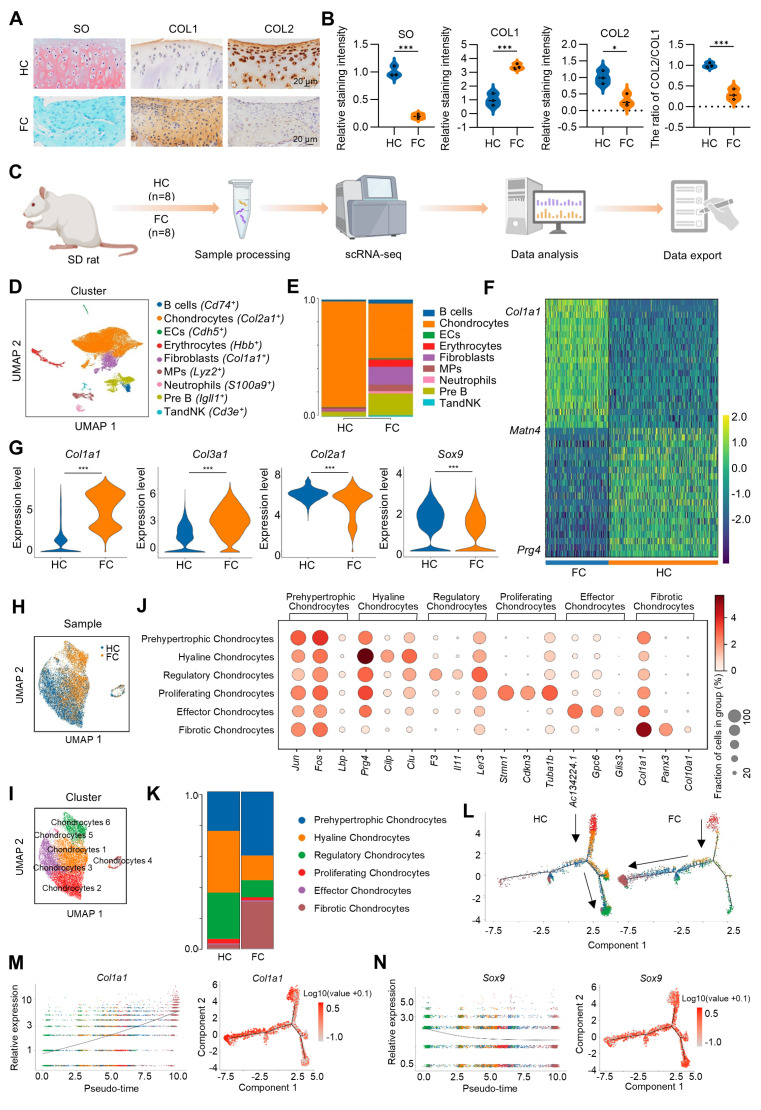
** scRNA-seq analysis of hyaline cartilage and fibrocartilage.** (A) Safranin O/fast green staining and immunohistochemical staining for COL1 and COL2 of rat hyaline cartilage and fibrocartilage, scale bar = 20 μm. (B) Quantification of SO, COL1 and COL2 staining intensity, and the ratio of COL2/COL1 (n = 3 per group). (C) Schematic of the scRNA-seq workflow. (D) Visualization of all cell types by Uniform Manifold Approximation and Projection (UMAP) plot, which was divided into 9 subpopulations. (E) Bar chart showing the distribution of different cell subclusters in HC and FC. (F) Heatmap of the scaled expression of top marker genes for HC and FC. (G) Violin plots showing the expression of *Col1a1, Col3a1, Col2a1* and *Sox9* in chondrocyte subpopulations in HC and FC. (H-I) Visualization of chondrocytes with UMAP plot, which was divided into 6 subclusters. (J) Dot plot of the scaled expression of top marker genes for different chondrocyte subpopulations. (K) The proportions of 6 chondrocyte subpopulations in HC and FC. (L) Pseudo-time analysis of chondrocyte subpopulations in HC and FC, colored by subcluster. (M-N) Pseudo-time differentiation trajectory analysis of the gene expression levels of *Col1a1* and *Sox9* during fibrocartilage formation. HC: hyaline cartilage, FC: fibrocartilage.

To investigate the pathophysiological mechanism of cartilage fibrosis and explore the potential molecular targets, an osteochondral injury was constructed in rat patella groove. This osteochondral injury model, similar to the bone marrow stimulation technique used in clinical practice, would initiate endogenous MSCs derived from bone marrow to spontaneously form fibrocartilage, which is composed of a large number of fibrotic chondrocytes highly expressing COL1 and reduced number of physiological hyaline chondrocytes expressing COL2 (Figure 1A-B). scRNA-seq was initially performed on the rat spontaneously formed fibrocartilage following osteochondral injury and rat normal hyaline cartilage with a comprehensive analysis of the heterogeneous cell subpopulations between these two groups (Figure 1C). Through cluster analysis of 25,789 cells obtained from hyaline cartilage (HC) and fibrocartilage (FC) samples of eight rats, we identified and annotated nine distinct cell types, including chondrocytes, fibroblasts, B cells, Pre B, mononuclear phagocytes (MPs), vascular endothelial cells (ECs), TandNK, neutrophils and erythrocytes (Figure 1D). Notably, chondrocytes constituted the predominant cell type in both HC and FC (Figure 1E), suggesting their crucial role in the regenerative process. Compared to the HC group, the FC group exhibited a substantial up-regulation of the fibrotic chondrocyte marker *Col1a1*, along with a marked down-regulation of *Prg4*, a marker for superficial zone chondrocytes, is highly expressed in superficial zone chondrocytes and chondroprogenitor cells 29-31 (Figure 1F). The violin plot further demonstrated that, in comparison to HC group, the expression of *Col1a1* and *Col3a1* was significantly up-regulated, while the expression of *Col2a1* and *Sox9* was significantly down-regulated in FC of chondrocyte subpopulations (Figure 1G). Furthermore, we extracted chondrocytes individually and performed re-clustering (Figure 1H-I). Based on the top expressed genes, chondrocytes were further divided into six cell subpopulations, including prehypertrophic chondrocytes (*Jun^+^*), hyaline chondrocytes (*Prg4^+^*), regulatory chondrocytes (*Il11^+^*), proliferating chondrocytes (*Cdkn3^+^*), effector chondrocytes (*Gpc6^+^*) and fibrotic chondrocyte (*Col1a1^+^*) (Figure 1J). To further analyze gene expression profiles, *Col2a1* was highly expressed across all chondrocyte subpopulations, indicating a chondrogenic phenotype (Figure S1). *Matn4* and *Prg4* were highly expressed in the hyaline chondrocyte subpopulation but showed reduced expression in the fibrotic chondrocyte subpopulation. Conversely, the fibrotic chondrocyte markers* Col1a1* and *Col3a1* were predominantly expressed in fibrotic chondrocyte subpopulation (Figure S1). Additionally, hyaline chondrocytes and regulatory chondrocytes constituted a larger proportion of HC, while fibrotic chondrocytes and prehypertrophic chondrocytes were more prevalent in FC (Figure 1K). These findings confirm the fibrocartilaginous nature of the spontaneously repaired tissue following osteochondral injury.

Subsequently, to elucidate the interrelationships among these chondrocyte subpopulations, we employed Monocle to construct a transcriptional trajectory of chondrocytes on a pseudo-time scale (Figure 1L, Figure S2). Based on the distinct biological functions identified for the six chondrocyte clusters, proliferating chondrocytes served as the starting point of differentiation, while regulatory chondrocytes and fibrotic chondrocyte represented the end stages of the differentiation process. Furthermore, a direct comparison between the HC and FC groups revealed that, in normal hyaline cartilage, proliferating chondrocytes predominantly differentiated into regulatory and hyaline chondrocytes. However, in fibrocartilage, proliferating chondrocytes exhibited a tendency to differentiate into fibrotic chondrocytes (Figure 1L). Consistent with these results, during fibrocartilage formation, the expression of the hyaline chondrocyte marker *Sox9* and *Prg4* progressively decrease (Figure 1N, Figure S3), while the expression of the fibrotic chondrocyte marker* Col1a1* was continuously up-regulated throughout the differentiation process (Figure 1M, Figure S3). Additionally, cell-cell communication analysis indicated that the interaction between chondrocytes and fibroblasts was the strongest, regardless of whether it was hyaline cartilage or fibrocartilage (Figure S4A-D).

**Figure 2:**
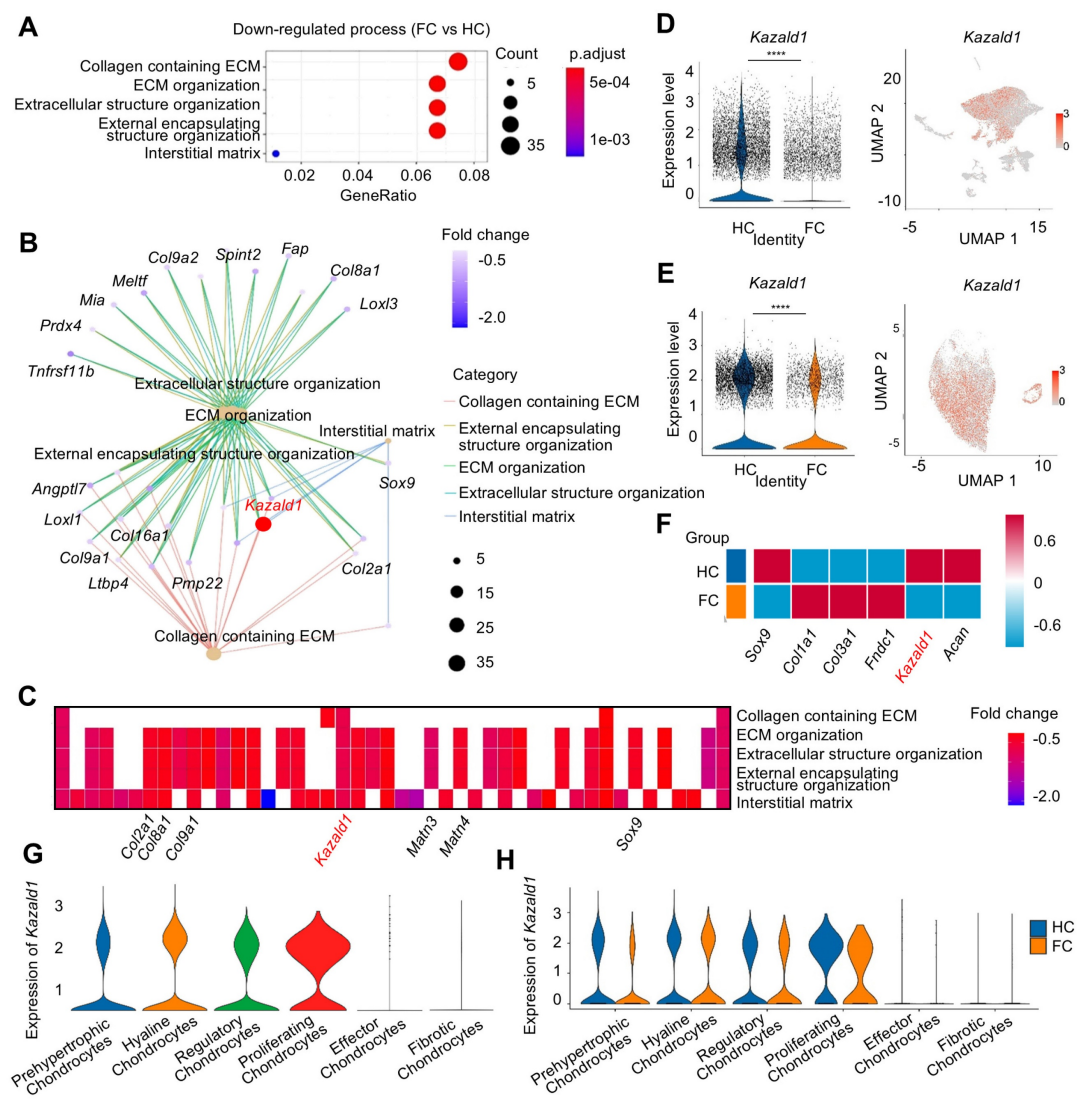
** The expression of Kazald1 is down-regulated in fibrocartilage following cartilage injury.** (A) Dot plot showing the significantly down-regulated biological processes in FC versus HC identified by GO enrichment analysis. (B) Key genes significantly enriched for the down-regulated GO terms between FC and HC. (C) Heatmap of the highly expressed genes enriched for top GO terms. (D) Violin plot and UMAP showing the expression of *Kazald1* in overall cell subpopulations in HC and FC. (E) Violin plot and UMAP showing the expression of *Kazald1* in chondrocyte subpopulations in HC and FC. (F) Heatmap of differentially expressed genes between HC and FC. (G) Violin plots showing *Kazald1* expression between different chondrocyte subclusters. (H) Violin plots showing *Kazald1* expression in different chondrocyte subclusters between HC and FC. HC: hyaline cartilage, FC: fibrocartilage. ****P < 0.0001.

To identify potential molecular targets involved in the regulation of cartilage fibrosis, GO analysis was conducted comparing the HC and FC groups. Compared to HC group, FC exhibited significant down-regulation of biological processes related to ECM remodeling, such as “Collagen containing ECM”, “ECM organization”, and “Extracellular structure organization” (Figure 2A), indicating the altered ECM of fibrocartilage. Several key hyaline cartilage markers, such as *Sox9, Col2a1* and *Col9a1*, were significantly enriched in these down-regulated GO terms between the FC and HC groups (Figure 2B). Among these core enrichment genes, *Kazald1* was also identified, indicating that it was notably down-regulated during fibrocartilage formation (Figure 2B). A heatmap of the top five GO terms for biological processes revealed that* Kazald1* is highly correlated with ECM assembly (Figure 2C). Consistently, violin plots demonstrated a significant decrease in *Kazald1* expression in fibrocartilage, both across the overall cell populations and specifically within chondrocyte subpopulations (Figure 2D-E).

To investigate whether Kazald1 contributed to fibrocartilage formation observed in cartilage injuries, we evaluated the expression of *Kazald1* along with typical hyaline cartilage markers (*Sox9* and *Acan*) and fibrocartilage markers (*Col1a1, Col3a1* and *Fndc1*). In the FC group compared to the HC group, *Kazald1*,* Sox9* and* Acan* expression was markedly suppressed, while *Col1a1, Col3a1* and *Fndc1* expression was substantially enhanced (Figure 2F). Among the six chondrocyte subclusters, *Kazald1* was highly expressed in the hyaline chondrocyte subcluster and nearly absent in the fibrotic chondrocyte subcluster (Figure 2G). In the comparison between FC and HC, the expression of *Kazald1* was down-regulated in almost all chondrocyte subpopulations (Figure 2H). Additionally, existing database of sequencing data on human musculoskeletal system revealed that *Kazald1* is highly expressed in normal human cartilage compared to bone, synovium or other musculoskeletal system tissues (Figure S5). Taken together, these findings suggest that Kazald1 plays a crucial role in maintaining cartilage homeostasis, and its down-regulation may contribute to the formation of fibrocartilage in response to cartilage injuries.

**Figure 3:**
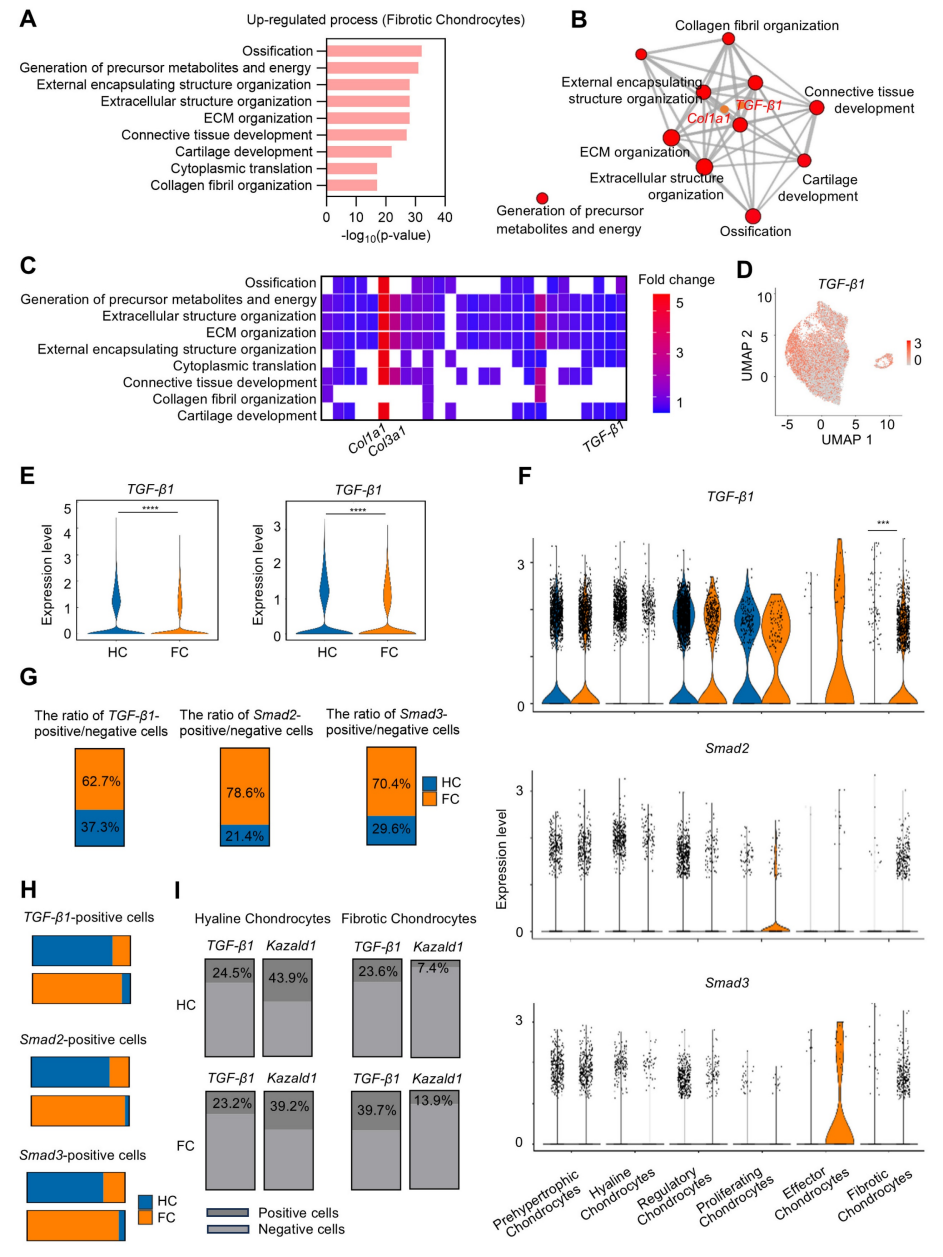
** The expression of TGF-β1 and Kazald1 becomes imbalanced during cartilage fibrosis.** (A) The significant up-regulated biological processes in fibrotic chondrocytes identified by GO enrichment analysis. (B) *TGF-β1* and *Col1a1* are significantly enriched for the up-regulated GO terms in fibrotic chondrocytes. (C) Heatmap of the highly expressed genes enriched for top GO terms. (D) UMAP showing the expression of *TGF-β1*. (E) Violin plots showing the expression of *TGF-β1* in overall cell subpopulations and chondrocyte subpopulations in HC and FC. (F) Violin plots showing the expression of* TGF-β1, Smad2,* and *Smad3* in different chondrocyte subclusters between HC and FC. (G) The ratio of *TGF-β1/Smad2/Smad3*-positive/negative cells in 6 chondrocyte subpopulations between HC and FC. (H) The *TGF-β1/Smad2/Smad3*-positive cells in hyaline chondrocytes and fibrotic chondrocytes between HC and FC. (I) The ratio of *TGF-β1*-positive and *Kazald1*-positive cells in fibrotic chondrocytes and hyaline chondrocytes in HC and FC. HC: hyaline cartilage, FC: fibrocartilage. ***P < 0.001, ****P < 0.0001.

The fibrotic chondrocyte subcluster plays a significant role in the initiation of fibrocartilage formation following cartilage injury. To better characterize the functional profiles of these fibrotic chondrocytes, GO analysis was performed, revealing that the FC group significantly up-regulated several biological processes related to ECM remodeling within this subcluster, including "Ossification," "External encapsulating structure organization," "ECM organization," and "Collagen fibril organization," compared to the HC group (Figure 3A). Notably, *Col1a1* was enriched among the up-regulated GO terms, suggesting its high expression in fibrotic chondrocytes. Furthermore, *TGF-β1*, a well-known pro-fibrotic inducer, was also enriched in these GO terms in the fibrotic chondrocyte subcluster (Figure 3B). A heatmap of the top GO terms for biological processes further demonstrated the strong expression of the fibrocartilage markers *Col1a1* and *Col3a1*, as well as *TGF-β1* in the fibrotic chondrocyte cluster (Figure 3C). Additionally, UMAP plot revealed that *TGF-β1* is expressed across six chondrocyte clusters (Figure 3D). Following cartilage injury, *TGF-β1* expression significantly decreased in FC relative to HC, both in overall cell populations and specifically within the chondrocyte clusters (Figure 3E), consistent with previous findings 13, 14. Upon further investigation of the six chondrocyte subpopulations, it was found that while *TGF-β1* expression decreased in most clusters in the FC group, it was notably elevated in the effector and fibrotic chondrocyte subclusters compared to the HC group (Figure 3F).

However, due to the limited number of effector chondrocytes, subsequent analyses focused primarily on the fibrotic chondrocyte subcluster. There was also a significant increase in the downstream transcription factors *Smad2* and *Smad3*, key components of the TGF-β1 signaling pathway, in the fibrotic chondrocyte subcluster of the FC group compared to the HC group (Figure 3F). Correspondingly, the proportion of cells positive/negative for *TGF-β1, Smad2,* and *Smad3* markedly increased in the fibrotic chondrocytes of the FC group compared to the HC group (Figure 3G). In the hyaline chondrocyte subcluster, the HC group exhibited a higher number of *TGF-β1* and *Smad2/3*-positive cells compared to the FC group. Conversely, in fibrotic chondrocytes, the FC group showed a significantly greater number of these positive cells than the HC group (Figure 3H). These findings further indicate the activation of the TGF-β1 signaling pathway in fibrocartilage.

We conducted a detailed analysis of the expression kinetics and correlation between *TGF-β1* and *Kazald1* in the hyaline and fibrotic chondrocyte subclusters of both HC and FC groups (Figure 3I). In the hyaline chondrocyte subpopulation, the proportion of *Kazald1*-positive cells was markedly higher than that of *TGF-β1*-positive cells in both the HC and FC groups. Conversely, in the fibrotic chondrocyte subpopulation, the proportion of *Kazald1*-positive cells was significantly lower than that of *TGF-β1*-positive cells across both groups. Direct comparison between HC and FC groups further revealed unique expression kinetics of *Kazald1* and *TGF-β1*. In the hyaline chondrocyte subpopulation, there was no significant change in *Kazald1-* or *TGF-β1-*positive cells between HC and FC groups. However, in the fibrotic chondrocyte subpopulation, *TGF-β1*-positive cells exhibited a marked increase in the FC group compared to the HC group, potentially driving cartilage fibrosis. Although there was a slight increase in the proportion of *Kazald1-*positive cells in the fibrotic chondrocyte subpopulation from HC to FC, its expression remained extremely low, nearly undetectable, and this modest increase was insufficient to exert any biological effect. Collectively, these analyses indicate that Kazald1 is a key regulatory molecule in maintaining cartilage homeostasis, where the balance between Kazald1 and TGF-β1 appears to direct the fate of chondrocytes. Specifically, higher Kazald1 levels compared to TGF-β1 favor the hyaline chondrocyte phenotype, while higher TGF-β1 levels compared to Kazald1 promote the fibrotic chondrocyte phenotype. Following cartilage injury, TGF-β1 levels increased significantly in the fibrotic chondrocyte subpopulation, while Kazald1 expression was markedly reduced and barely detectable, preventing Kazald1 from counteracting the pro-fibrotic effects of TGF-β1.

### Recombinant Kazald1 protein attenuates chondrocyte fibrosis and facilitates chondrocyte phenotype maintenance *in vitro*

**Figure 4:**
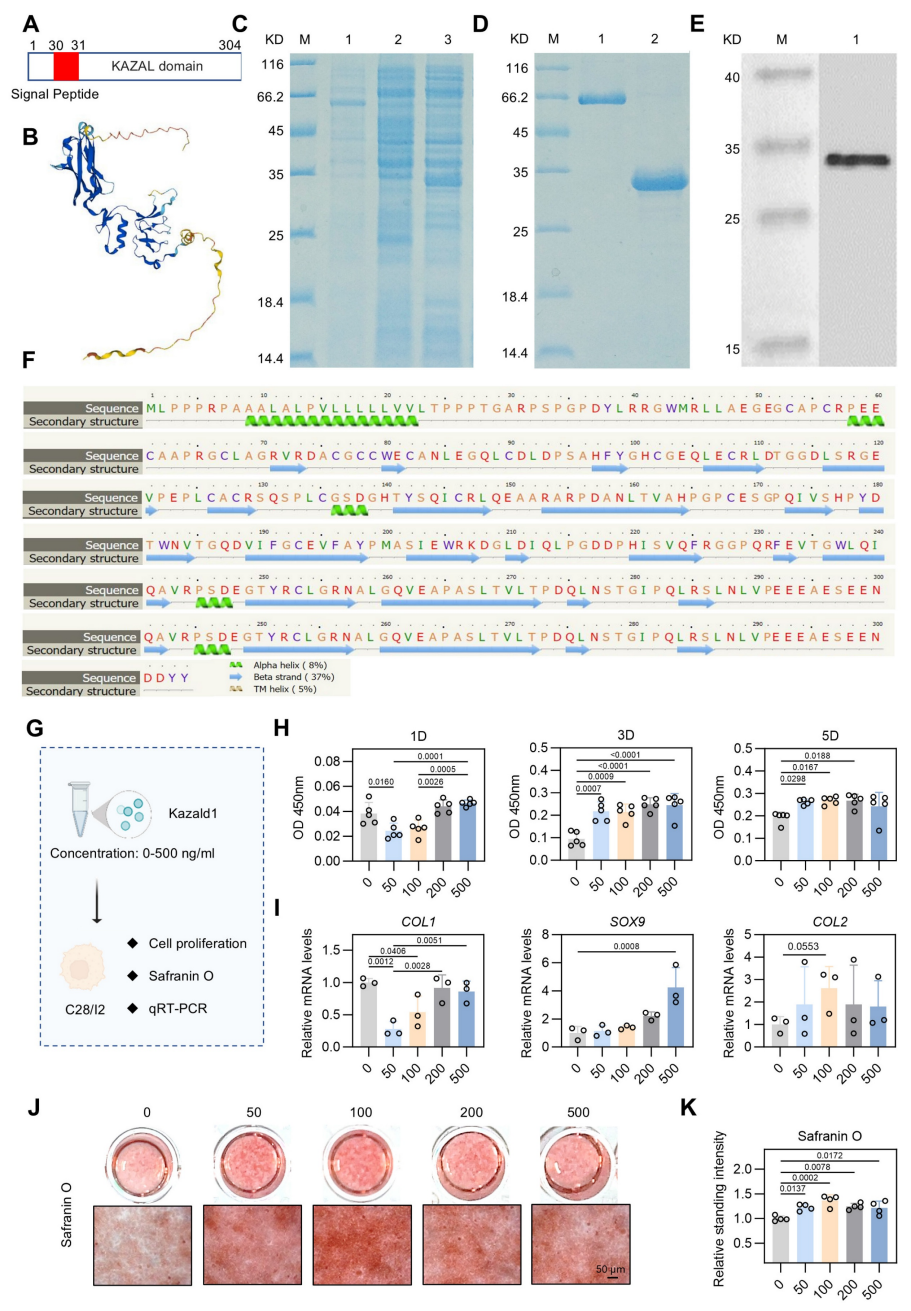
** Kazald1 inhibits chondrocyte fibrosis and preserves chondrocyte phenotype in human chondrocytes.** (A) The molecular structure of human Kazald1 predicted by bioinformatic analysis based on Uniprot (https://www.uniprot.org/), showing a signal peptide (aa 1-30), and a Kazal domain (aa 31-304). (B) Tertiary structure of Kazald1 predicted by AlphaFold web portal (https://alphafold.ebi.ac.uk/). (C) SDS-PAGE showing the human Kazal domain (aa 31-304) expressed in bacteria, BL21 (DE3). Lane 1, empty vector pET30a; Lane 2, pET-30a-Kazald1 (uninduced); Lane 3, pET-30a-Kazald1 (induced). (D) SDS-PAGE showing the purified recombinant His-tagged human Kazald1 protein. Lane 1, BSA; lane 2, purified recombinant human Kazald1 protein. (E) Western blot showing that recombinant His-tagged human Kazald1 protein probed with anti-His antibody. (F) Secondary structure of Kazald1 predicted by the Phyre2 web portal (http://www.sbg.bio.ic.ac.uk/phyre2/). (G) Schematic of *in vitro* cell experiments to assess the effect of Kazald1 on human C28 chondrocytes. (H) CCK-8 assay evaluating the proliferation of human chondrocytes treated with different concentrations of Kazald1 protein on days 1, 3, and 5 (n = 5 per group). (I) Gene expression levels of fibrocartilage marker *COL1* and hyaline cartilage markers* SOX9* and* COMP* on day 3 (n = 3 per group). (J-K) Safranin O staining and quantification for human chondrocytes treated with different concentrations of Kazald1 protein on day 7 (n = 4 per group), scale bar = 50 μm. Results are shown as mean ± SD.

Through scRNA-seq analysis, we hypothesized that supplementing Kazald1 may inhibit chondrocyte fibrosis and facilitate the maintenance of healthy chondrocyte phenotype. However, previous research on Kazald1 is limited, and no studies have investigated its effects on chondrocyte fibrosis or fibrocartilage formation, either through recombinant protein or gene transfer strategies. Kazald1 consists of three distinct domains: an N-terminal insulin growth factor-binding domain, a central Kazal-type serine protease inhibitor and follistatin-like domain, and a C-terminal immunoglobulin-like domain 23. The presence of multiple domains highlights its potential multifunctionality and implicates its involvement in various physiological processes. In this study, we expressed and purified the full length of recombinant human Kazald1 protein (aa 31-304) (Figure 4A-D), with predicted tertiary and secondary structures (Figure 4E-F). Subsequent experiments were performed to determine whether the recombinant Kazald1 protein could inhibit chondrocyte fibrosis and preserve chondrocyte phenotype in *in vitro* cultured chondrocytes.

First, to determine the optimal concentration of recombinant Kazald1 protein for cell growth, we cultured human C28 chondrocytes with varying concentrations of Kazald1 ranging from 0-500 ng/ml (Figure 4G). Despite a decrease in cell proliferation at 50 ng/ml on day 1, CCK-8 assay revealed that all concentrations of recombinant Kazald1 substantially enhanced cell proliferation on days 3 and 5 compared to the 0 ng/ml group, indicating its good cytocompatibility (Figure 4H). To further investigate the effect of Kazald1 on chondrocyte phenotype maintenance, we examined the expression of key cartilage-related genes by qPCR. Compared to the 0, 200, and 500 ng/ml groups, 50 and 100 ng/ml substantially down-regulated the expression of the fibrocartilage marker *COL1* (Figure 4I). Besides, compared to the 0 ng/ml group, treatment with 500 ng/ml Kazald1 protein significantly increased the expression of* SOX9* without affecting the expression of* COL2*. The 100 ng/ml concentrations showed a mild, but not significant, up-regulation of both *SOX9* and *COL2* (Figure 4I). Safranin O staining showed that the addition of recombinant Kazald1 protein significantly enhanced GAG deposition in human chondrocytes after 7 days of culture, and 100 ng/ml had the most significant effect (Figure 4J-K). Additionally, alcian blue staining demonstrated that concentrations of 50, 100, and 200 ng/ml significantly enhanced GAG deposition on day 7 compared to the 0 ng/ml group (Figure S7A-C). The heatmap for systematic dose-effect relationship analysis demonstrated that 100 ng/ml Kazald1 most effectively maintained the chondrocyte phenotype (*SOX9, COL2* expression) and promote GAG production (Safranin O staining), while simultaneously inhibited chondrocyte fibrosis (*COL1* expression) (Figure S6).

Kazald1 is highly conserved across multiple species 25. Therefore, we proceeded to assess the effect of recombinant Kazald1 protein on mouse ATDC5 chondrocytes (Figure S8A). CCK-8 assay showed that treatment with recombinant Kazald1 protein at concentrations ranging from 50-500 ng/ml promoted early proliferation of ATDC5 chondrocytes on day 3 (Figure S8B). qPCR results indicated that 100 and 200 ng/ml concentrations of Kazald1 significantly promoted *COL2* expression and slightly increased *SOX9* expression (Figure S8C). From the results observed in both human and mouse chondrocytes, the 100 ng/ml concentration of recombinant Kazald1 protein exhibited a mild but significant effect in supporting the maintenance of chondrocyte phenotype and inhibiting chondrocyte fibrosis. Consequently, we selected 100 ng/ml recombinant Kazald1 protein for subsequent experiments.

### Kazald1 effectively inhibits TGF-β1-induced chondrocyte fibrosis to preserve chondrocyte phenotype

**Figure 5:**
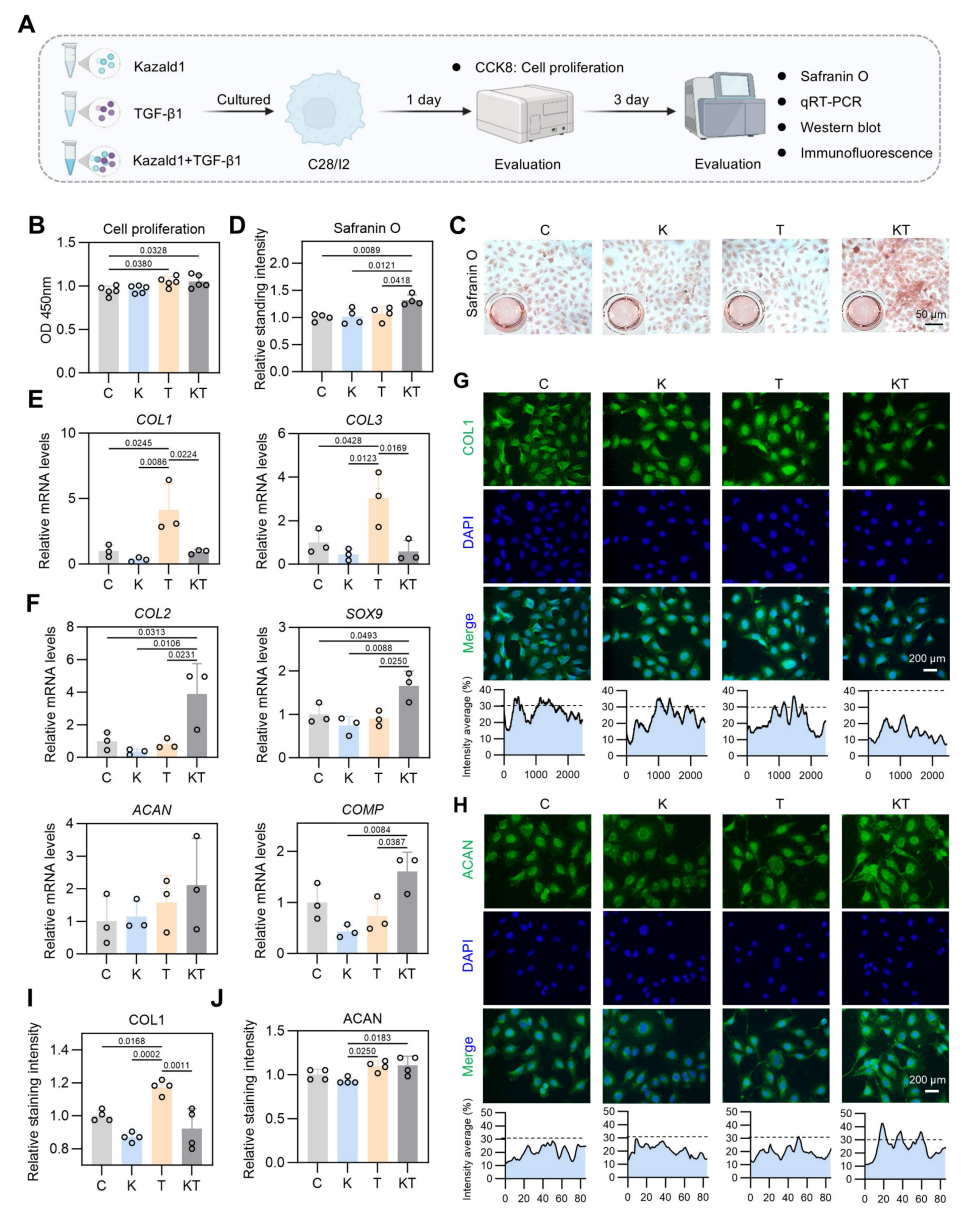
** Kazald1 inhibits TGF-β1-induced chondrocyte fibrosis to preserve chondrocyte phenotype.** (A) Schematic of *in vitro* cell experiments to assess the synergistic effect of Kazald1 and TGF-β1 on human C28 chondrocytes. (B) CCK-8 assay evaluating the proliferation of human chondrocytes treated with Kazald1, TGF-β1 or Kazald1+TGF-β1 on day 1 (n = 5 per group). (C-D) Safranin O staining and quantification for human chondrocytes treated with Kazald1, TGF-β1 or Kazald1+TGF-β1 on day 3 (n = 4 per group). (E) Gene expression levels of *COL1* and* COL3* on day 3 (n = 3 per group). (F) Gene expression levels of *COL2*, *SOX9, ACAN,* and* COMP* on day 3 (n = 3 per group). (G-H) Immunofluorescent staining for COL1 and ACAN on day 3, scale bar = 200 μm. (I-J) Quantification of immunofluorescent staining (n = 4 per group). Results are shown as mean ± SD.

TGF-β1 is a well-recognized key factor in cartilage development and maintenance 32, 33. However, it often initiates a temporary collagen accumulation program that accelerates cartilage fibrosis 34, limiting its widespread application in the clinical treatment of cartilage injuries. Our scRNA-seq results revealed a significant increase in the proportion of fibrotic chondrocytes following cartilage injury, likely induced by the activated TGF-β1 pathway in this subcluster. Notably, the expression of Kazald1 is significantly down-regulated, preventing it from counteracting the pro-fibrotic TGF-β1 signaling. This ultimately leads to chondrocyte fibrosis and fibrocartilage formation. After evaluating the effect of recombinant Kazald1 protein on chondrocyte fibrosis and determining the optimal concentration, we proceeded to test whether the recombinant Kazald1 protein could inhibit TGF-β1-induced chondrocyte fibrosis and boost the therapeutic potential of TGF-β1 (Figure 5A).

We initially conducted a CCK-8 assay to assess cell proliferation with Kazald1 and/or TGF-β1 treatment, which showed that both the TGF-β1 and Kazald1+TGF-β1 groups significantly enhanced human chondrocyte proliferation compared to the control group on day 1 (Figure 5B). Safranin O staining revealed that the Kazald1+TGF-β1 group promoted greater GAG deposition compared to the other groups (Figure 5C-D). Additionally, Alcian blue staining demonstrated that while Kazald1 or TGF-β1 individually led to a modest enhancement of GAG secretion, their combined treatment significantly increased GAG deposition, as indicated by the strongest staining intensity observed (Figure S9A-B). As expected, TGF-β1 significantly elevated the expression levels of fibrotic chondrocyte marker genes, including *COL1* and* COL3* (Figure 5E), confirming its pro-fibrotic effect. Importantly, the up-regulation of these fibrotic genes induced by TGF-β1 was effectively inhibited by the synergistic supplementation of recombinant Kazald1 protein (Figure 5E). Meanwhile, the Kazald1+TGF-β1 group significantly promoted the expression of hyaline chondrocyte marker genes, including *COL2*, *SOX9*, *ACAN,* and *COMP* compared to the control, Kazald1, and TGF-β1 groups (Figure 5F). These results were further validated at the protein level. Immunofluorescent staining revealed that TGF-β1 substantially elevated the production of COL1, while Kazald1 significantly abrogated the TGF-β1-induced up-regulation of this fibrotic chondrocyte marker (Figure 5G, I). The protein expression of ACAN exhibited a mild but significant increase with TGF-β1 or Kazald1+TGF-β1 treatment (Figure 5H, J).

In addition to human chondrocytes, we confirmed the potential of Kazald1 to counteract TGF-β1-induced fibrosis in rat chondrocytes (Figure S10A). Consistent with the findings in human chondrocytes, TGF-β1 strongly promoted *COL1* gene expression, which was significantly inhibited by the synergistic treatment of Kazald1 (Figure S10B). Immunofluorescent staining further demonstrated that TGF-β1 notably increased COL1 expression, which was significantly reduced by the addition of Kazald1, resulting in an expression level even lower than that in the control group (Figure S11A-B). Furthermore, the TGF-β1 group and Kazald1+TGF-β1 group exhibited a significant up-regulation of *COL2* expression compared to the control group (Figure S10B). Collectively, these findings indicate that recombinant Kazald1 protein effectively inhibits TGF-β1-induced chondrocyte fibrosis, thereby favoring a hyaline chondrocyte phenotype.

### Mechanisms underlying the inhibitory effect of Kazald1 on TGF-β1-induced chondrocyte fibrosis

We subsequently explored the mechanisms by which Kazald1 inhibits TGF-β1-induced chondrocyte fibrosis. The pro-fibrotic TGF-β1 signaling pathway is initiated when the TGF-β1 ligand binds to its receptors (TGFBR), leading to the phosphorylation of downstream targets Smad2 and Smad3. These phosphorylated Smad then translocated into the nucleus, where they regulate the transcription of fibrotic genes Smad2 and Smad3, which are structurally similar, often act synergistically to mediate TGF-β1 signaling 35. Additionally, TGF-β1 induces other signaling pathways, including the activation of the phosphatidylinositol 3-kinase (PI3K)/Akt pathway to promote various types of tissue/organ fibrosis, such as pulmonary fibrosis 36-38. However, it remains unclear whether the TGF-β1/Akt/Smad signaling pathways are involved in chondrocyte fibrosis and whether Kazald1 directly interferes with these pathways to inhibit chondrocyte fibrosis.

**Figure 6:**
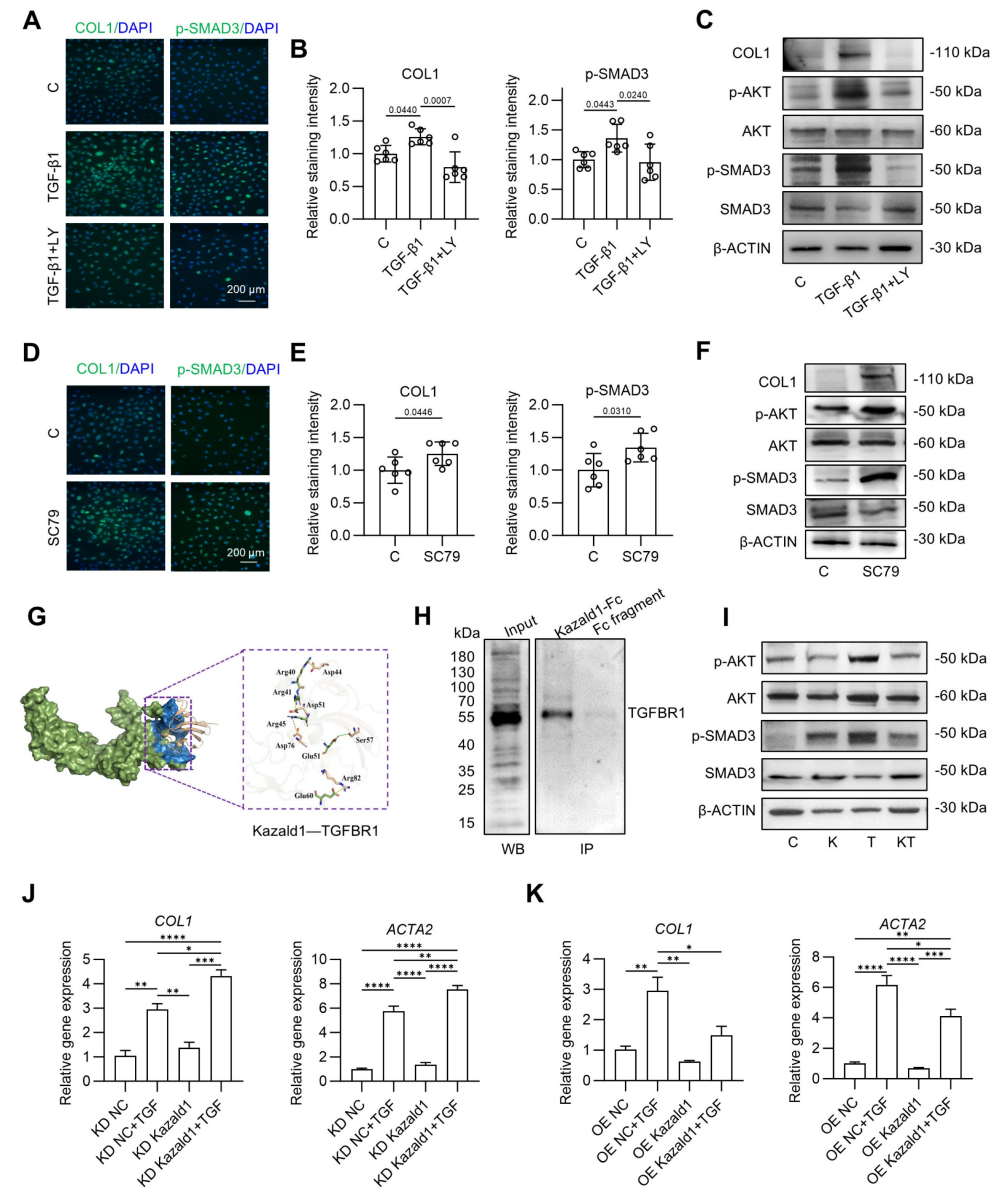
** Mechanisms underlying the inhibitory effect of Kazald1 on TGF-β1-induced chondrocyte fibrosis.** (A-B) Immunofluorescent staining and quantification for COL1 and p-Smad3 after treatment with TGF-β1 with/without LY2109761 on day 1 (n = 6 per group), scale bar = 200 μm. (C) Western blot for COL1, p-AKT, AKT, p-SMAD3, and SMAD3 after treatment with TGF-β1 with/without LY2109761 on day 1. (D-E) Immunofluorescent staining and quantification for COL1 and p-Smad3 after treatment with SC79 on day 1 (n = 6 per group), scale bar = 200 μm. (F) Western blot for COL1, p-AKT, AKT, p-SMAD3, and SMAD3 after treatment with SC79 on day 1. (G) Molecular docking between Kazald1 and TGFBR1. The TGFBR1 is presented in a surface model (smudge), with its extracellular domain highlighted in blue. The Kazald1 is illustrated in a wheat-colored cartoon model. The yellow dashed lines indicate the salt bridge action, and the green dashed lines indicate the hydrogen bond action. (H) Co-IP assay of Kazald1 and TGFBR1. (I) Western blot for p-AKT, AKT, p-SMAD3, and SMAD3 after treatment with Kazald1, TGF-β1 or Kazald1+TGF-β1 on day 1. (J-K) Gene expression levels of *COL1* and *COL3* in human chondrocytes treated with shKazald1 or AAV-shKazald1 virus, with or without TGF-β1, on day 3 (n = 3 per group). Results are shown as mean ± SD. *P<0.05, **P<0.01, ***P < 0.001, ****P < 0.0001.

To address this, we first examined whether the activation of the TGF-β1/Akt/Smad signaling could induce chondrocyte fibrosis. Immunofluorescent staining and Western bolt analysis showed that treatment with TGF-β1 led to increased phosphorylation of both AKT and SMAD3, along with an up-regulation in COL1 expression (Figure 6A-C). These effects were effectively reversed by LY2109761, a selective inhibitor of TGFBR 39. Co-treatment with LY2109761 and TGF-β1 significantly inhibited TGF-β1-induced AKT and SMAD3 phosphorylation, consequently suppressing COL1 expression and mitigating chondrocyte fibrosis compared to the TGF-β1 treated group (Figure 6A-C). To further investigate the relationship between Akt pathway and chondrocyte fibrosis, we utilized SC79, an Akt activator 40, to stimulate chondrocytes. SC79 treatment not only significantly increased AKT phosphorylation but also enhanced SMAD3 phosphorylation and COL1 expression (Figure 6D-F), in a manner consistent with TGF-β1 induction. Together, these findings suggest that TGF-β1 induces phosphorylation of AKT and SMAD3, with activated AKT further phosphorylating SMAD3, resulting in the up-regulated expression of the fibrotic chondrocyte marker COL1.

Next, we investigated how Kazald1 inhibits TGF-β1-induced chondrocyte fibrosis. Since the pro-fibrotic TGF-β1/Akt/Smad signaling is activated by the binding of TGF-β1 to TGFBR1, we first explored whether Kazald1 interfered with this interaction. Molecular docking analysis revealed that the N-terminal of Kazald1 is closely bind to the extracellular region of TGFBR1 (Figure 6G). At interaction interface, Arg40, Arg41 and Arg45 of Kazald1 form a salt bridge with Asp44, Asp51 and Asp76 of TGFBR1, respectively. Besides, The Glu51 of Kazald1 forms a hydrogen bond with Ser57 of TGFBR1, while the Glu60 of Kazald1 forms a salt bridge with Arg82 of TGFBR1. These interactions can efficiently anchor and stabilize the Kazald1-TGFBR1 complex (Figure 6G). This protein-protein interactions were also validated by Co-IP, which demonstrated the binding between Kazald1 and TGFBR1 (Figure 6H). In addition, the immunoprecipitation-mass spectrometry (IP-MS) results indicated that, when searching for transmembrane proteins among the top ten proteins with the highest binding affinity to Kazald1, TGFBR1 was also identified (Figure S12). These results suggest that Kazald1 forms a dimer with TGFBR1, thereby hindering the binding of TGF-β1 and TGFBR1, potentially disrupting the downstream pro-fibrotic pathway activated by TGF-β1. To test this hypothesis, we assessed downstream Akt and Smad3 activation following treatment with Kazald1 and TGF-β1. Compared to the control and Kazald1 groups, TGF-β1 group significantly increased AKT phosphorylation (Figure 6I). However, simultaneous treatment with Kazald1 and TGF-β1 resulted in a significant reduction in p-AKT levels, indicating that Kazald1 effectively inhibited TGF-β1-induced AKT phosphorylation. Furthermore, compared to the control group, treatment with TGF-β1 alone resulted in up-regulation of p-SMAD3 in chondrocytes (Figure 6I). Conversely, concurrent treatment with both factors significantly reduced p-SMAD3 expression, demonstrating that Kazald1 effectively mitigated TGF-β1-induced SMAD3 phosphorylation. Collectively, the interaction of Kazald1 with TGFBR1 effectively blocks downstream Akt/Smad3 signaling, thereby inhibiting TGF-β1-induced chondrocyte fibrosis.

To further investigate the role of Kazald1 on TGF-β1-induced chondrocytes fibrosis, we conducted both Kazald1 knockdown and overexpression experiments. Kazald1 was silenced using short hairpin RNA (shKazald1, KD Kazald1) and overexpressed via an AAV-shKazald1 vector (OE Kazald1). Transfection with the shRNA-expressing virus, shKazald1 and AAV-shKazald1, led to a clear reduction and increase in Kazald1 expression, respectively (Figure S13). qPCR results showed no significant changes in fibrotic genes following Kazald1 knockdown or overexpression. However, after Kazald1 knockdown followed by TGF-β1 treatment (KD Kazald1+ TGF-β1), the expression of *COL1* and *ACTA2* was further up-regulated compared to KD NC+ TGF-β1 group (Figure 6J). Similarly, Kazald1 overexpression in the presence of TGF-β1 (OE Kazald1+ TGF-β1) significantly inhibited the expression of *COL1* and *ACTA2* as compared to OE NC+ TGF-β1 group (Figure 6K). This result further demonstrates that Kazald1 can alleviate TGF-β1-induced chondrocytes fibrosis.

### Kazald1 and TGF-β1 combination promotes structural and functional hyaline cartilage regeneration in rats

We further investigate whether recombinant Kazald1 protein, either alone or in combination with TGF-β1, could promote hyaline cartilage regeneration rather than fibrocartilage formation* in vivo*. Due to the rapid release and short retention of direct drug delivery, which would compromise therapeutic efficacy 41, a photo-crosslinked gelatin methacryloyl (GelMA)/silk fibroin methacryloyl (SilMA) composite hydrogel was developed to serve as a carrier for Kazald1 and TGF-β1, allowing controlled delivery while providing a compatible environment for cell ingrowth 42. GelMA hydrogels are widely used as delivery systems for various biomolecules due to their excellent biocompatibility and favorable physicochemical properties. However, GelMA alone exhibits rapid degradation, which present challenges for the sustained release of recombinant proteins *in vivo*. To address this, we incorporated SilMA into the hydrogel system to further prolong its degradation time, by establishing ester bonds between GelMA and SilMA (Figure S14). The GelMA, SilMA, and GelMA-SilMA composite hydrogels were photo-crosslinked under UV irradiation, with no significant difference in gelation time among the groups (Figure S15A-B). Scanning electron microscopy (SEM) revealed microporous and interconnected structures within these hydrogels (Figure S16A). The average pore size and porosity of the GelMA-SilMA hydrogel were measured at 66.95 µm and 90.97%, respectively, falling between those of GelMA (84.16 µm and 85.67%) and SilMA (50.40 µm and 92.43%) hydrogels (Figure S16B-C). Furthermore, the GelMA-SilMA hydrogel exhibited substantially prolonged degradation kinetics, with almost 30% remaining after 77 days, compared to the complete degradation of GelMA by 56 days (Figure S17). All hydrogels demonstrated good cytocompatibility with chondrocytes, showing minimal cell death and normal cell proliferation (Figure S18).

**Figure 7:**
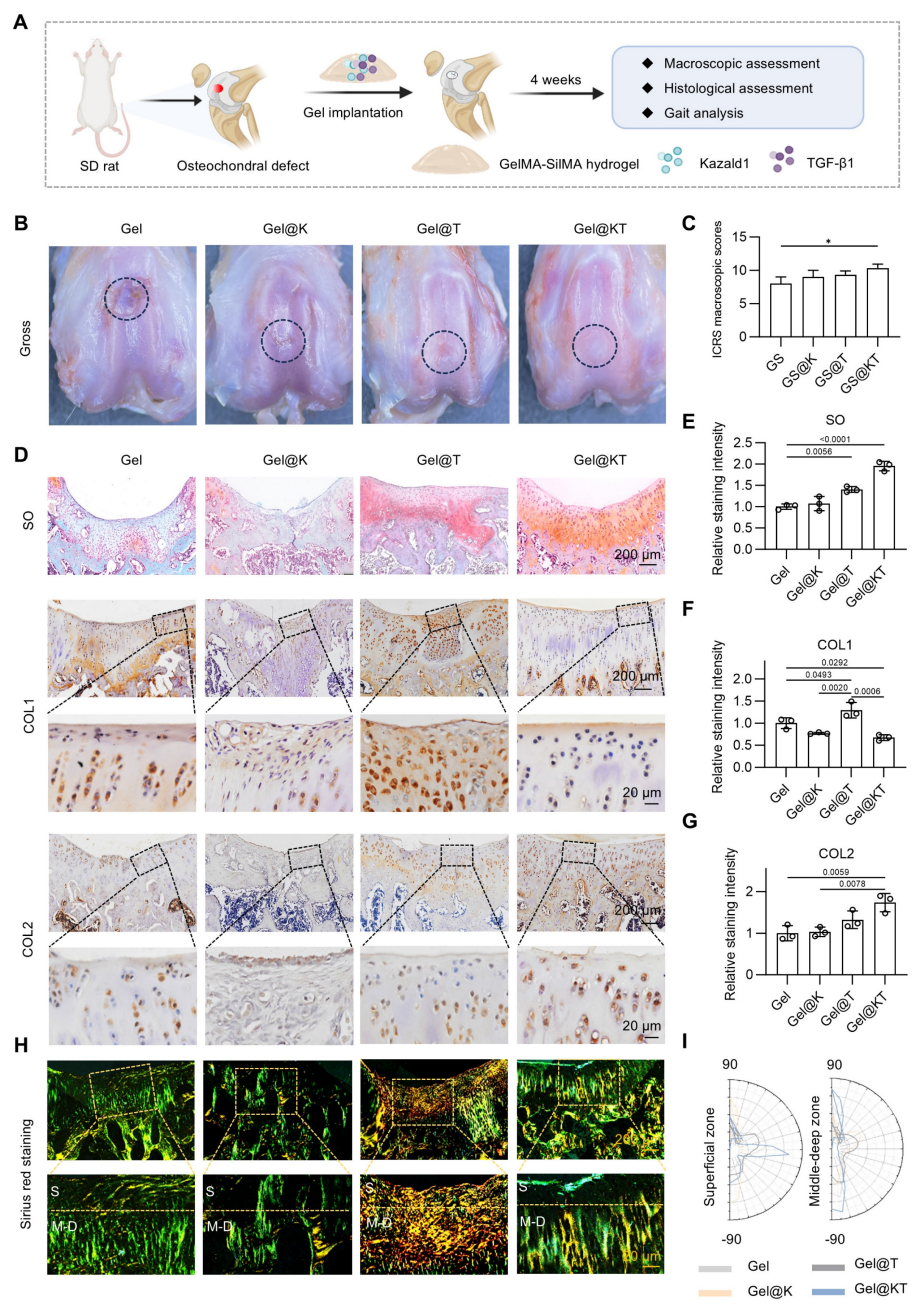
** Kazald1 and TGF-β1 combination promotes structural restoration of hyaline cartilage in rat prophylactic treatment model of osteochondral injuries.** (A) Schematic of the animal study procedure. Kazald1 and TGF-β1 were incorporated into GelMA-SilMA composite hydrogels, which were then implanted to treat rat osteochondral defects. (B) Macroscopic views of repaired cartilage treated with Gel, Gel@K, Gel@T, and Gel@KT hydrogels. (C) ICRS macroscopic scores of repaired cartilage. (D) Safranin O/fast green staining and immunohistochemical staining for COL1 and COL2 of repaired cartilage in Gel, Gel@K, Gel@T, and Gel@KT groups, scale bar = 200 μm/20 μm. (E) Quantification of safranin O/fast green staining (n = 3 per group). (F-G) Quantification of immunohistochemical staining for COL1 and COL2 (n = 3 per group). (H) Picrosirius red staining and polarized light microscopy of repaired cartilage, scale bar = 200 μm/20 μm. (I) Collagen fibers distribution in superficial zone (upper 1/3) and middle-deep zone (bottom 2/3) of repaired cartilage. Results are shown as mean ± SD. *P<0.05.

Next, we established a rat prophylactic treatment model of osteochondral injuries and delivered Kazald1 and/or TGF-β1 within the GelMA-SilMA hydrogel carrier *in situ* (Figure 7A). Four weeks post-operatively, specimens were collected for macroscopic, histological and functional evaluations. Gross morphology images revealed that, in the pure GelMA-SilMA hydrogel group (Gel), the cartilage defect area was obviously larger than in the other groups, with a clear cavity in the center (Figure 7B). The Kazald1-loaded (Gel@K) and TGF-β1-loaded (Gel@T) hydrogel groups showed increasing amounts of newly-formed white tissue filling the defect areas, though some cracks remained (Figure 7B). In contrast, the Kazald1 and TGF-β1-loaded hydrogel group (Gel@KT) exhibited nearly complete regeneration of the cartilage surface, characterized by glossy white, smooth, and well-integrated hyaline cartilage-like tissue (Figure 7B). Based on the ICRS scoring system, the overall repair assessment score for the Gel@KT group was the highest, with an average score of 10.33 ± 1.07, significantly higher than the other three groups (Figure 7C).

Histological analysis was performed using safranin O/fast green staining to assess ECM remodeling and cell phenotype (Figure 7D-E). Safranin O/fast green staining revealed an obvious depression in the repaired tissue of the Gel group, consisting mostly of fibrous tissue with minimal safranin O staining. The Gel@K group exhibited a large crack and a fibrous, disorganized ECM within the repaired tissue. Improved cartilage regeneration with enhanced GAG deposition was observed in the Gel@T group. However, the regenerated cartilage showed moderate fibrosis, as indicated by faintly stained ECM and a fibrotic chondrocyte phenotype. In contrast, the Gel@KT group exhibited smooth, well-integrated repaired tissue, with intense safranin O staining and typical chondrocyte phenotype indicating improved hyaline cartilage regeneration (Figure 7D-E). Moreover, immunohistochemistry for COL1 and COL2 was conducted to characterize the specific ECM proteins in the newly formed tissue (Figure 7D, F-G). Positive staining of COL1 was evident in the Gel group, confirming the fibrocartilage nature of the repaired tissue following osteochondral injuries. The Gel@T group also showed strong COL1 staining, reflecting the pro-fibrotic effect of TGF-β1 *in vivo*, consistent with the results observed *in vitro* (Figure 5). However, the intensity of COL1 staining in the Gel@K and Gel@KT groups was significantly decreased than in the Gel and Gel@T groups, indicating a notable inhibition of cartilage fibrosis. In contrast, the Gel@KT group showed the most intense COL2 staining in the regenerated cartilage, while relatively weak COL2 staining was observed in the Gel, Gel@K, and Gel@T groups (Figure 7D, 7G).

Cartilage fiber alignment was further evaluated to assess the quality of repaired tissue. Based on the native hyaline cartilage structure, two independent regions of interest (ROI) were selected to detect collagen fiber orientation: the superficial zone (S zone, upper 1/3) with typically horizontal alignment fibers, and the middle-deep zone (M-D zone, bottom 2/3) with vertical orientated fibers. This unique alignment may be attributed to the distinct functional roles of these cartilage regions: the superficial fibers facilitate cushioning and lubrication, while the fibers in the middle-deep zone grow vertically into the subchondral bone 43, 44. Picrosirius red staining revealed that the collagen structure of the repaired cartilage in the Gel@KT group was more organized than the other three groups (Figure 7H-I). Polar plot analysis further corroborated that the Gel@KT group demonstrated the most favorable collagen fiber alignment in both the superficial and middle-deep zone (Figure 7H-I).

**Figure 8:**
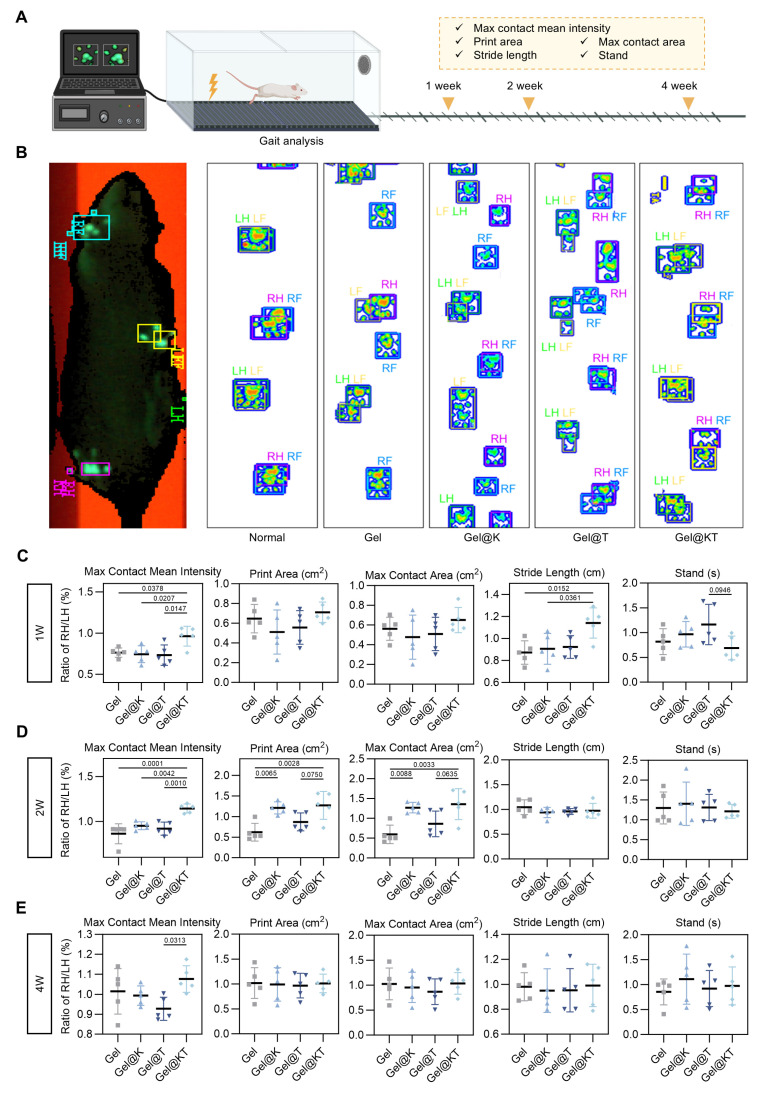
** Kazald1 and TGF-β1 combination promotes functional recovery of repaired cartilage.** (A) Gait analysis was performed at 1-, 2- and 4-weeks post-surgery. (B) Tracking of pawprints during walking in normal rats and four treatment groups post-surgery. (C-E) Representative gait parameters of rats among groups at 1-, 2- and 4-weeks post-surgery (n = 5 per group). Results are shown as mean ± SD. RF: right front, LF: left front, RH: right hind, LH: left hind.

To further evaluate the functional recovery of regenerated cartilage after different treatments *in vivo*, gait analysis was conducted at weeks 1, 2, and 4 post-operatively using the CatWalk XT^®^ system (Figure 8A). Gait analysis is a feasible and noninvasive approach to assess locomotor function through the evaluation of behavioral phenotypes. During walking, paw prints of the rats were captured using a real-time fluorescence imaging system, revealing distinct walking motion between groups (Figure 8B). In normal rats, the pawprints of ipsilateral front and hind limbs (i.e., LF and LH, RF and RH) almost overlapped. However, in the Gel, Gel@K, and Gel@T groups, the prints of the front limbs were not aligned with those of the ipsilateral hind limbs, indicating irregular walking motion. In contrast, the Gel@KT group exhibited well-ordered paw prints and improved balance, similar to that of the normal rats (Figure 8B).

Quantitative assessment of gait parameters was performed and compared among groups (Figure 8C-E). Poor cartilage regeneration and increased pain levels adversely affected the rats' walking motion, leading to a decrease in parameters related to print intensity and contact area, while an increase in parameters related to walking duration. The "max contact mean intensity," which measures the mean intensity at the maximum paw contact area during walking, was significantly higher in the Gel@KT group compared to the control, Gel@K, and Gel@T groups in the first and second weeks postoperatively. By the fourth week, the Gel@KT group continued to show a substantial increase in maximum contact mean intensity compared to the other groups and was significantly higher than the Gel@T group. This suggests that the combined treatment of Kazald1 and TGF-β1 effectively improved cartilage regeneration and mitigated postoperative pain. Both the Gel@K and Gel@KT groups showed enhancements in "print area" (surface area of the complete print) and "max contact area" (maximum area of a paw that comes into contact with the ground) compared to the other groups in the second week postoperatively. Additionally, regarding "stride length," the Gel@KT group was significantly higher than the other groups in the first week, indicative of reduced joint pain. For "standing time" (the duration of paw contact with the ground), the Gel@KT group had lower values than the Gel@T group in the first week, but showed no differences between other groups in the second and fourth weeks. Collectively, these results demonstrate that the Gel@KT group enhanced functional recovery of repaired cartilage by improving walking motion and alleviating pain.

Additionally, we evaluated the long-term efficacy and safety of Kazald1 on cartilage repair. At 12 weeks post-surgery, histological staining and Micro‑CT analyses demonstrated that the combination treatment of Kazald1 and TGF-β1 effectively maintained the hyaline cartilage phenotype over longer periods, without evident fibrosis (indicated by negative COL1 and positive COL2 staining in the regenerated cartilage) or heterotopic ossification (indicated by Micro‑CT images) (Figure S19).

**Figure 9:**
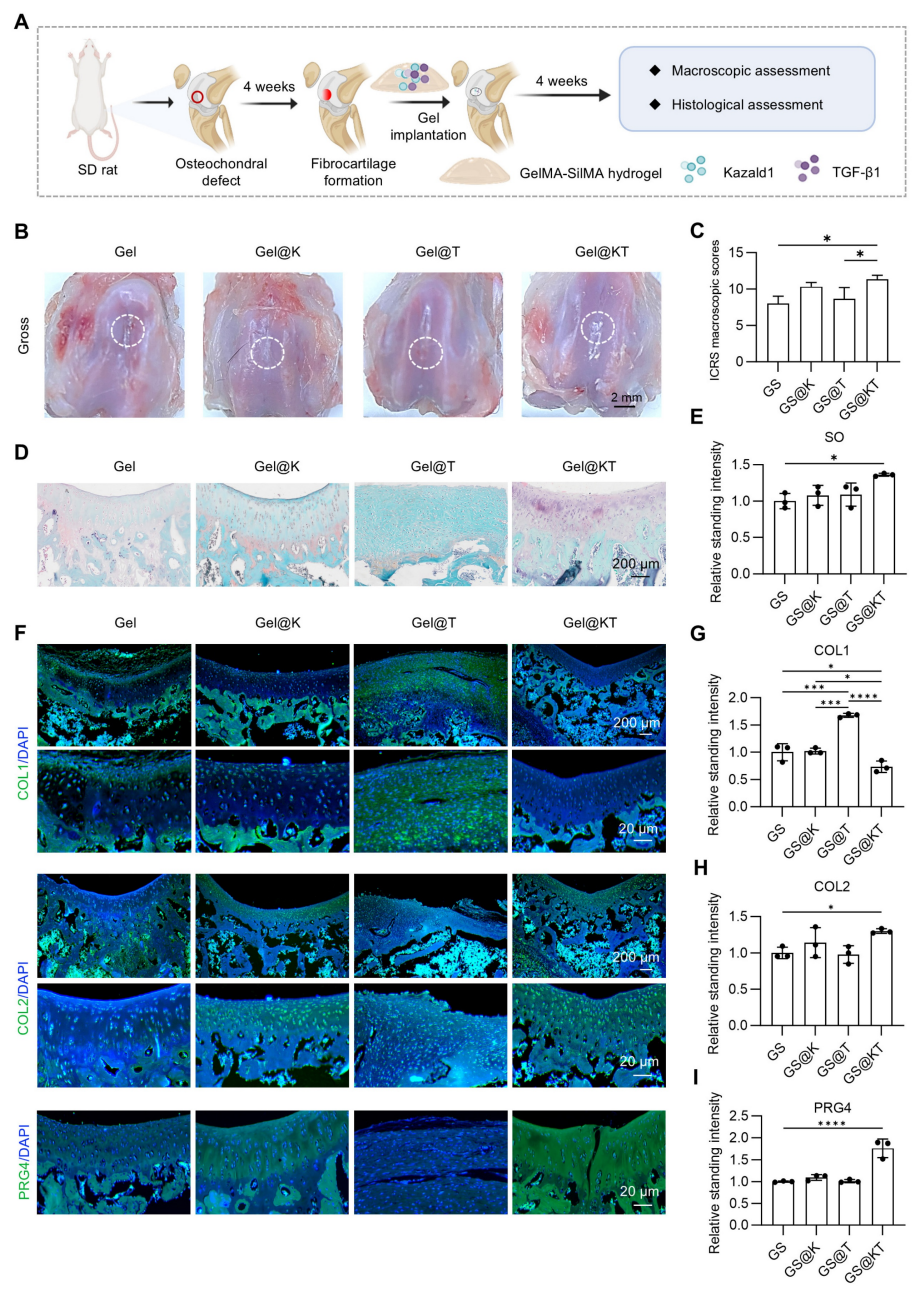
**Kazald1 and TGF-β1 combination reverses fibrocartilage formation in rat therapeutic treatment model of osteochondral injuries.** (A) Schematic of the animal study procedure. (B) Macroscopic views of repaired cartilage treated with Gel, Gel@K, Gel@T, and Gel@KT hydrogels. (C) ICRS macroscopic scores of repaired cartilage. (D) Safranin O/fast green staining. (E) Quantification of safranin O/fast green staining (n = 3 per group). (F) Immunofluorescent staining for COL1, COL2 and PRG4 of repaired cartilage in Gel, Gel@K, Gel@T, and Gel@KT groups, scale bar = 200 μm/20 μm. (G-I) Quantification of immunofluorescent staining for COL1, COL2 and PRG4 (n = 3 per group). Results are shown as mean ± SD. *P<0.05, ***P < 0.001, ****P < 0.0001.

While our results demonstrated that Kazald1 can promote hyaline cartilage regeneration in a prophylactic treatment model when administered in the initial stages of fibrosis onset, it remains unclear whether Kazald1 can reverse fibrocartilage into hyaline cartilage once fibrosis has already developed. Therefore, we established a therapeutic treatment model to evaluate the ability of Kazald1 to reverse fibrocartilage formation. In this model, Kazald1 was applied four weeks after surgery, followed by a four-week repair phase (Figure 9A). As shown in Figure 1A, fibrocartilage had formed by week 4 post-injury. After an additional 4 weeks of treatment (i.e., 8 weeks post-injury), the Gel@K and Gel@KT groups exhibited smoother and more intact surface filling compared to the control and Gel@T groups (Figure 9B). Furthermore, ICRS scoring indicated that the Gel@KT group achieved the highest scores among all groups, with values significantly higher than those of the Ctrl and Gel@T groups (Figure 9C). Histological analysis revealed organized cellular distribution and abundant GAGs in the cartilage defect area of the Gel@KT group (Figure 9D-E). In addition, repaired cartilage treated with Gel@KT showed decreased COL1 expression and increased COL2 and PRG4 expression compared to the other groups (Figure 9F-I). Collectively, these findings indicate that the Gel@KT hydrogel effectively promote the formation of hyaline cartilage over fibrocartilage in a rat therapeutic treatment model of osteochondral injuries.

Collectively, these findings indicate that the Gel@KT hydrogel effectively promotes the structural restoration of hyaline cartilage rather than fibrocartilage in rat osteochondral injuries.

### Kazald1 and TGF-β1 combination improves hyaline cartilage maintenance in human cartilage explants

**Figure 10:**
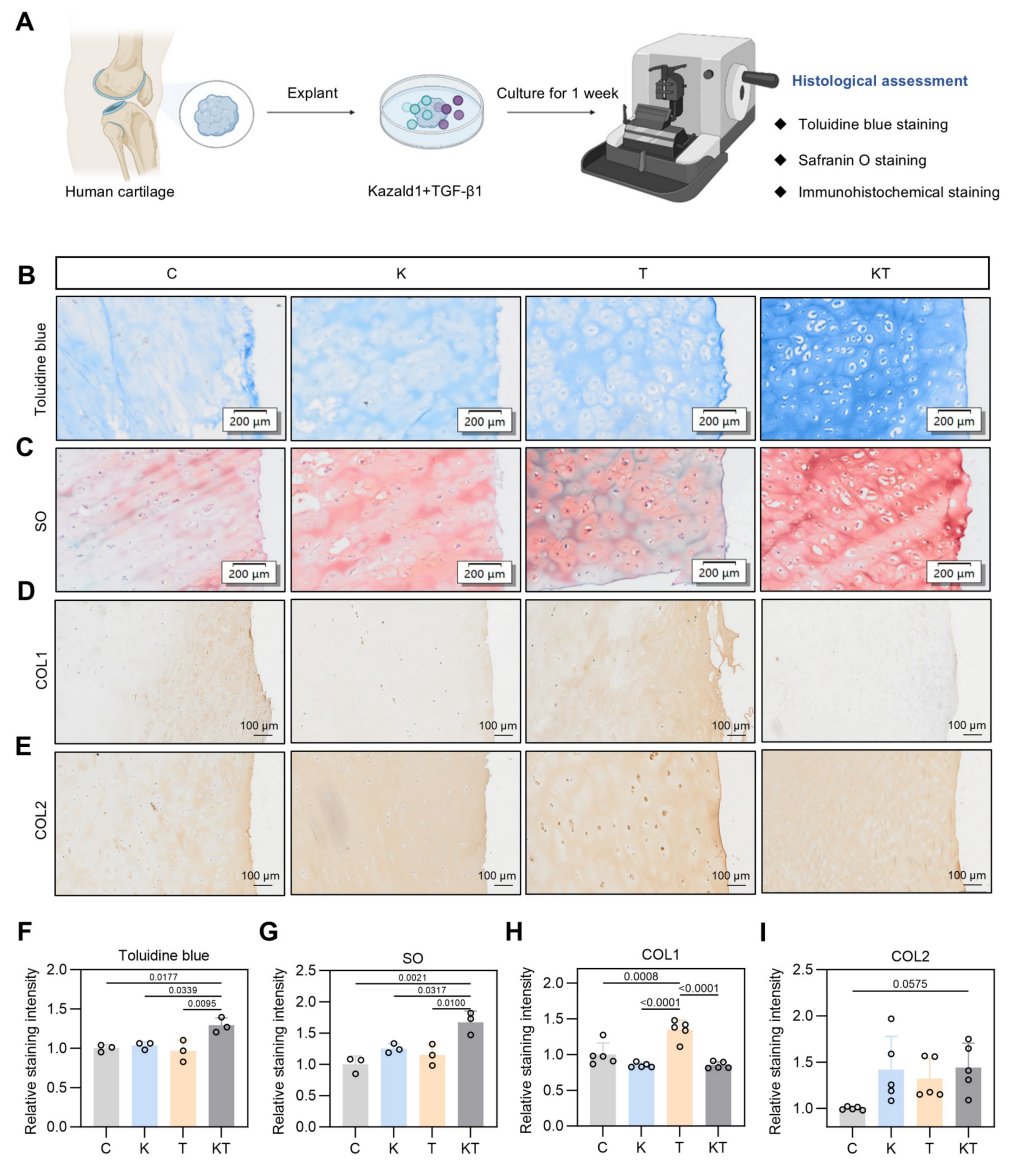
** Kazald1 and TGF-β1 combination improves hyaline cartilage maintenance in human cartilage explants.** (A) Schematic of the human cartilage explant culture procedure. (B-C) Toluidine blue staining and safranine O/fast green staining of cartilage explant treated with Kazald1, TGF-β1 or Kazald1+TGF-β1 on day 7, scale bar = 200 μm. (D-E) Immunohistochemical staining for COL1 and COL2, scale bar = 200 μm. (F-G) Quantification of toluidine blue staining and safranin O/fast green staining (n = 3 per group). (H-I) Quantification of immunohistochemical staining for COL1, COL2 (n = 5 per group). Results are shown as mean ± SD.

The positive effect of the Kazald1 and TGF-β1 combination in promoting hyaline cartilage regeneration in rat models prompted us to explore its potential in a clinical scenario. To determine whether this combination treatment could inhibit TGF-β1-induced cartilage fibrosis and improve hyaline cartilage maintenance in humans, we obtained cartilage explants from five OA patients and performed explant culture with Kazald1 and TGF-β1 treatments for one week (Figure 10A). Patient information is provided in Table S4. The cartilage tissue was harvested from the region adjacent to the OA-affected area that showed no obvious signs of degeneration. Toluidine blue and safranine O/fast green staining revealed a predominantly rounded, typical hyaline chondrocyte phenotype in the explants treated with the Kazald1 and TGF-β1 combination, whereas a mix of fibrotic and hyaline chondrocyte phenotypes was observed in the control, Kazald1 alone, and TGF-β1 alone groups (Figure 10B-C). The combination of Kazald1 and TGF-β1 significantly enhanced the staining intensity compared to the other three groups, indicating increased cartilaginous GAG secretion (Figure 10B-C, 10F-G). Immunohistochemical staining for COL1 revealed that compared to the control group, the TGF-β1 group notably increased COL1 expression in cartilage explants, verifying the pro-fibrotic effect TGF-β1 in human. This increase in COL1 expression was subsequently reduced by the synergistic treatment of Kazald1 and TGF-β1 to a level even lower than that of the control group (Figure 10D, Figure 10H). Additionally, the Kazald1+TGF-β1 group exhibited significantly higher COL2 expression, compared to the control or the Kazald1 group (Figure 10E, Figure 10I). These findings further support the beneficial effects of the Kazald1 and TGF-β1 combination in attenuating TGF-β1-induced cartilage fibrosis and improving hyaline cartilage maintenance in human cartilage explants.

## Discussion

Once articular cartilage is damaged, its inherent repair potential is limited. Clinically, while repair tissue does form, it often comprises mechanically inferior fibrocartilage rather than the desired hyaline cartilage. However, there is still a lack of effective clinical strategies to inhibit fibrocartilage formation and enhance hyaline cartilage regeneration following cartilage injury. Kazald1, a secreted member of the IGFBP superfamily, has been associated in limited studies with glioma progression, limb regeneration, and osteoblast proliferation 24. In this study, we identified Kazald1 for the first time as a potential therapeutic target for mitigating cartilage fibrosis by counteracting the pro-fibrotic effect of TGF-β1. The combination of Kazald1 and TGF-β1 significantly reduced the expression of fibrotic markers, such as COL1 and COL3, while promoting the expression of hyaline chondrocyte markers, including COL2 and SOX9, thereby favoring a more favorable chondrocyte phenotype. Furthermore, we validated the therapeutic effect of this Kazald1 and TGF-β1 combination in promoting hyaline cartilage regeneration and maintenance using rat osteochondral injury models and human cartilage explants, respectively.

TGF-β, a vital factor involved in cartilage physiology and pathology, plays a dual role by promoting chondrogenic differentiation while also inducing chondrocyte fibrosis 45, 46. Previous studies have demonstrated that physiological doses of TGF-β (0.1-1 ng/ml) promote the formation of engineered cartilage exhibiting naive mechanical properties and chondrocyte phenotype. However, engineered cartilage cultured with super-physiological doses of TGF-β (10-100 ng/ml) has been observed to exhibit phenotypic characteristics indicative of chondrocyte fibrosis and hypertrophy 47. In articular cartilage, TGF-β is highly expressed in healthy cartilage to maintain chondrocyte homeostasis but is significantly down-regulated following cartilage injury or disease 13, 14. This raises an intriguing question: if TGF-β is abundantly expressed in normal hyaline cartilage without causing fibrosis, why does its down-regulation after injury trigger fibrocartilage formation? We hypothesize the existence of specific regulatory molecules that modulate the pro-fibrotic effects of TGF-β in health and injured cartilage. Through the first scRNA-seq analysis of hyaline cartilage and fibrocartilage samples, we found that following cartilage injures, TGF-β1 expression is significantly down-regulated in fibrocartilage compared to hyaline cartilage, consistent with previous findings 10, 11.

However, at the single-cell level, different chondrocyte subpopulations exhibited varying TGF-β1 expression patterns: it decreased in the hyaline chondrocyte subpopulation while it substantially increased in the fibrotic chondrocyte subpopulation within fibrocartilage, indicating the dual role of TGF-β1 in directing the cell fate of different subclusters toward fibrotic or hyaline phenotypes based on different expression levels. Within specific chondrocyte subpopulations, Kazald1 is highly expressed in hyaline chondrocyte subcluster but is nearly absent in the fibrotic chondrocyte subpopulation, both in health and injured cartilage. Further analysis of expression kinetics and the correlation between TGF-β1 and Kazald1 revealed that in the hyaline chondrocyte subpopulation, the proportion of Kazald1-positive cells is markedly higher than that of TGF-β1-positive cells, suggesting that Kazald1 can counteract the pro-fibrotic effects of TGF-β1, thus maintaining the hyaline chondrocyte phenotype. Conversely, in the fibrotic chondrocyte subpopulation, the proportion of Kazald1-positive cells is significantly lower than that of TGF-β1-positive cells, indicating that Kazald1 is unable to counteract TGF-β1's pro-fibrotic effects, leading to a fibrotic phenotype. Following cartilage injury, the significant increase in TGF-β1 within the fibrotic chondrocyte subpopulation, coupled with the substantial decrease in Kazald1, prevents Kazald1 from counteracting the pro-fibrotic effects of TGF-β1, thereby exacerbating chondrocyte fibrosis and inducing fibrocartilage formation. Thus, Kazald1 emerges as a key regulator of TGF-β1, with expression patterns of both TGF-β1 and Kazald1 differing across chondrocyte subpopulations and between healthy and fibrocartilage states. The balance between TGF-β1 and Kazald1 ultimately determines chondrocyte fate, and up-regulation of Kazald1 could prevail over pro-fibrotic TGF-β1 signaling, favoring a normal chondrocyte phenotype and enhancing hyaline cartilage regeneration.

Through scRNA-seq analysis, it is logical to hypothesize that the supplementation of exogeneous Kazald1 following cartilage injury could potentially ameliorate fibrocartilage formation. However, to date, no studies have reported such interventions, whether through recombinant protein delivery or gene transfer strategies. In this study, we expressed and purified recombinant human Kazald1 protein, which contains the predicted functional Kazal domain (aa 31-304), and evaluated its therapeutic potential against chondrocyte fibrosis and fibrocartilage formation both *in vitro* and *in vivo*. Our results suggested that Kazald1 exerted a dose-dependent effect, with 100 ng/ml demonstrating a mild effect on chondrocyte proliferation, GAG production and fibrosis inhibition. A more pronounced effect was observed when Kazald1 was combined with TGF-β1, resulting in significant inhibition of chondrocyte fibrosis and preservation of the hyaline chondrocyte phenotype. This is attributed to the fact that, in the absence of TGF-β1 stimulation, chondrocytes maintain a healthy state with minimal fibrosis. Conversely, exogenous TGF-β1 particularly at the super-physiological concentration used in this study (10 ng/ml)-induces a fibrotic chondrocyte phenotype, and Kazald1 exhibits a more pronounced effect in this stimulated condition by inhibiting the pro-fibrotic effect of TGF-β1. We speculated that Kazald1, which binds to TGFBR1 and inhibits the interaction between TGF-β1 and its receptor, effectively reduces the working concentration of TGF-β1. This allowed TGF-β1 to act in a more physiological range, thereby exerting pro-chondrogenic effects. Thus, the combination of Kazald1 and TGF-β1 effectively inhibits chondrocyte fibrosis while maintaining a hyaline chondrocyte phenotype. The therapeutic efficacy of this combination was subsequently validated in rat prophylactic and therapeutic treatment models of osteochondral injuries and in human cartilage explant culture studies. Both studies demonstrated that the Kazald1 and TGF-β1 combination significantly suppressed fibrocartilage formation and enhanced hyaline cartilage regeneration and maintenance.

Tissue and organ fibrosis is a significant cause of global morbidity and mortality, prompting numerous preclinical and clinical studies aimed at identifying anti-fibrotic drugs that target the aberrant signaling pathways involved in fibrosis across various tissues and organs, including the liver, heart, kidneys, and lungs 48. Among these efforts, targeting the TGF-β pathway has emerged as the most extensively investigated approach for inhibiting fibrosis, given that TGF-β cytokines play a central role in most fibrotic processes. For example, Pirfenidone, one of the two FDA-approved drugs for idiopathic pulmonary fibrosis (IPF), inhibits both the synthesis and activation of TGF-βs, resulting in reduced lung function decline, decreased mortality, and improved overall survival for IPF patients 49, 50. However, cartilage fibrosis and fibrocartilage formation have often been overlooked, with only a limited number of studies reporting on anti-cartilage fibrosis drugs. For instance, Liu et al. demonstrated that the RNA helicase DDX5 can inhibit hyaline cartilage fibrosis and degradation in osteoarthritis through mechanisms involving pre-mRNA splicing and G4 unfolding in chondrocytes 51. Li et al. suggested that microtubule-targeting agents, such as docetaxel, may promote the transition from fibrocartilage to hyaline cartilage by modulating microtubule stability 8. Despite these findings, there have been no studies exploring pharmacological interventions targeting TGF-β signaling specifically for inhibiting cartilage fibrosis. Here, we identified Kazald1 as a novel key regulator capable of inhibiting chondrocyte fibrosis by interfering with TGF-β1 signaling through dimer formation with TGF-β1 receptor. This interaction suppresses the phosphorylation of downstream Akt and Smad3, ultimately inhibiting the expression of fibrotic genes. Importantly, we did not observe any serious adverse effects associated with Kazald1 during the evaluation period, which have been reported with previous pharmacological approaches aimed at inhibiting TGF-β signaling 52, 53. Therefore, this therapeutic strategy with Kazald1 protein presents a promising intervention for cartilage fibrosis, contributing novel insights to the field that remains largely underexplored in current research.

This study has several limitations that warrant consideration: 1) Our single-cell RNA sequencing of the repaired cartilage revealed that among the TGF-β isoforms (TGF-β1, TGF-β2, and TGF-β3), TGF-β1 showed both high expression levels in chondrocyte subpopulations and the most pronounced differential expression between hyaline cartilage and fibrocartilage, whereas TGF-β2 and TGF-β3 remained at quite low levels. This suggests that TGF-β1 may play a significant role in fibrocartilage formation following cartilage injury, leading us to select TGF-β1 for subsequent investigations. However, whether Kazald1 also interacts with other TGF-β isoforms remains unclear and will be explored in future studies. 2) In addition to the synergistic effect of Kazald1 and TGF-β1, we also noted that Kazald1 alone has a mild effect on inhibiting fibrosis and maintaining hyaline chondrocyte phenotype. This effect may be attributed to the inhibition of endogenous TGF-β1 by Kazald1 or to a specific anti-fibrotic pathway initiated by Kazald1 that functions independently of TGF-β1 signaling. Future studies are warranted to explore the specific receptors for Kazald1 and elucidate its downstream regulatory mechanisms.

## Conclusion

In conclusion, this study unveils the pivotal role of Kazald1 in the regulation of cartilage fibrosis and highlights its potential as a therapeutic agent for facilitating hyaline cartilage regeneration. Kazald1 functions by interfering pro-fibrotic TGF-β1 signaling through the formation of dimer with TGF-β1 receptor, leading to the inactivation of downstream Akt and Smad3 pathways and the subsequent suppression of fibrotic gene expression. The combination treatment of Kazald1 with TGF-β1 markedly inhibits chondrocyte fibrosis and fibrocartilage formation, while effectively facilitating hyaline cartilage regeneration and maintenance in both animal models and human cartilage explants. This study enhances our understanding of the regulatory mechanisms underlying cartilage fibrosis and proposes a viable therapeutic strategy for enhanced hyaline cartilage regeneration.

## Supplementary Material

Supplementary materials and methods, figures and tables.

## Figures and Tables

**Table 1:** Primer sequences

Gene	Species	Forward	Reverse
*GAPDH*	Human	TGACGCTGGGGCTGGCATTG	GGCTGGTGGTCCAGGGGTCT
*COL2*	Human	GTCTGTGACACTGGGACTGT	TCTCCGAAGGGGATCTCAGG
*SOX9*	Human	GGCGGAGGAAGTCGGTGAAGAA	GCTCATGCCGGAGGAGGAGTGT
*ACAN*	Human	CTGCAGACCAGGAGGTATGTGA	GTTGGGGCGCCAGTTCTCAAAT
*COMP*	Human	AACAGTGCCCAGGAGGAC	TTGTCTACCACCTTGTCTGC
*COL1*	Human	CGATGGATTCCAGTTCGAGTAT	CATCGACAGTGACGCTGTAGG
*COL3*	Human	TTTTGCAGTGATATGTGATGTT	GGATGGTGGTTTTCAGTTTA
