# Supplementary material for: Kazald1 attenuates chondrocyte fibrosis to potentiate hyaline cartilage regeneration by interfering with the pro-fibrotic TGF-β signaling
Source: Theranostics. 2025 Sep 8;15(18):9885–910. doi: 10.7150/thno.113604 (PMC12486400; doi:10.7150/thno.113604)
Supplement: Supplementary file 1 — Supplementary materials and methods, figures and tables. [file thnov15p9885s1.pdf]

Supporting Information

Kazald1 attenuates chondrocyte fibrosis to potentiate hyaline cartilage regeneration by interfering with the pro-fibrotic TGF-β signaling

Yue Zhu[a, 1], Haoyang Liu[a, 1], Yuting Zhang[b, 1], Danning Zheng[a], Yaning Li[b], Jialin Chen[a, c, d], Jiake Xu[b, e, *], Wei Zhang[a, d]**

[a] School of Medicine, Jiangsu Key Laboratory for Biomaterials and Devices, Southeast University, Nanjing, 210000, China.

[b] Shenzhen University of Advanced Technology, and Shenzhen Institutes of Advanced Technology, Chinese Academy of Sciences, Shenzhen, 518055, China.

[c] Department of Ophthalmology, Zhongda Hospital, Southeast University, 210009, Nanjing, China.

[d] China Orthopedic Regenerative Medicine Group (CORMed), 310058, Hangzhou, China.

[e] School of Biomedical Sciences, The University of Western Australia, Perth, Western Australia, 6000, Australia.

Materials and methods:

Rat chondrocyte isolation and culture

SD rats were sacrificed and subjected to strict disinfection procedures. The cartilage tissue located on the metaphysis of the knee joint was excised and rinsed twice with PBS. After washing, the cartilage tissues were cut into small pieces and incubated in H-DMEM supplemented with 200 U/ml type II collagenase (Biofroxx, Germany) at 37 °C in a 5% CO_2 atmosphere overnight. The resulting cell suspension was centrifuged at 1200 rpm for 10 min, resuspended in H-DMEM supplemented with 10% FBS and 1% P/S, and then subjected to standard cell culture procedures. Chondrocytes with 3-5 passages were utilized for the following study.

Alcian blue staining

Chondrocytes were cultured in 96-well plates with a growth medium supplemented with Kazald1 and/or TGF-β1 for 3 and 7 days. Alcian blue staining (1%, pH = 2.5, Macklin, China) was used to assess the level of cartilaginous GAG production. Following photography, staining was eluted with 6 M guanidine hydrochloride (Sinopharm, China), and the eluted dye solution was measured at 630 nm wavelength with a microplate reader (BioTek, USA).

Immunoprecipitation-mass spectrometry (IP-MS)

ATDC5 cells were harvested using the Pierce™ GPCR Membrane Protein Extraction Kit (A43436). Subsequently, the protein concentration was quantified via the BCA assay. Recombinant Kazald1-Fc protein (1 μg) and Fc fragment (1 μg) were individually pre-incubated with 10 μL of protein A/G magnetic beads (ThermoFisher Scientific, 88802) overnight at 4 °C. Next, 1 mg of total proteins were added into each sample tube, followed by a 1 h incubation at room temperature. After incubation, the beads were washed three times with PBS containing 0.05% Tween-20, followed by non-denatured elution for protein identification by mass spectrometry. The eluted proteins were reduced with 10mM DTT and alkylated with 55 mM IAA, and then digested using FASP strategy at 37 °C overnight. Peptides were desalted using C18 column and analyzed using timsTOF Pro2 (Bruker Daltonics) mass spectrometer and UHPLC system (Bruker Daltonics). Spectronaut (version 18.0) was used for data processing. The binding proteins of Kazald1 was identified by subtracting the binding proteins of the Fc fragment and then ranking the candidate proteins according to the MS intensity response.

Preparation of hydrogel carrier

A 10% (w/v) solution of GelMA (Engineering for Life, China), SilMA (Xingyue Biotechnology, China), or a 1:1 mixture of GelMA and SilMA was prepared. A photo-crosslinker, LAP (40 mg/mL, Engineering for Life, China), was subsequently mixed with the hydrogel precursor in a ratio of 40:1 at 37 °C. When indicated, recombinant Kazald1 protein (100 ng/ml) and/or Recombinant TGF-β1 protein (10 ng/mL, PeproTech, USA) were added to the GelMA-SilMA solution. The mixture underwent UV irradiation (wavelength: 365–370 nm; light intensity: 50 mW cm^{-2}) for 20 s to facilitate the photocrosslinking of the GelMA-SilMA hydrogels.

Scanning electron microscope (SEM) imaging

The hydrogels were freeze-dried for 24 h, sputter-coated with gold and imaged under SEM (Zeiss supra55, Germany) to observe the microstructure of the scaffold surface.

Pore size and porosity

The pore size of the freeze-dried hydrogels was analyzed with ImageJ software (NIH, USA) using SEM images. The porosity was calculated with the ethanol-exchange method. The dried hydrogel was weighed (Wh) and then immersed in anhydrous ethanol to obtain the wet weight (Wa). The porosity of hydrogel was calculated using the following equation:

Porosity = Wa - Wh/ρV. ρ represents the density of ethanol, and V is the volume of the dried hydrogel.

***In vitro* degradation**

The *in vitro* degradation of hydrogels was evaluated by incubating the samples in PBS at pH 7.4 and 37 °C with gentle shaking for a duration of 35 days. At each predetermined time point, the hydrogels

were retrieved from the solution, rinsed with distilled water, and subsequently dried in an oven. The initial dry weight (W_1) and the dry weight post-degradation (W_2) were recorded. The percentage of weight remaining was calculated using the following formula:

Weight remaining (%) = (W_2 / W_1) × 100.

Long-term animal study

SD rats (200 ± 20 g) were anesthetized with sodium pentobarbital by intraperitoneal injection at a dosage of 50 mg/kg. Subsequently, a cylindrical osteochondral defect (2 mm in height and 2 mm in diameter) was created in the right patellar groove, and Gel@KT hydrogel was injected into the defect site, followed by UV-initiated gelation. The operated animals were sacrificed 12 weeks post-surgery, and the cartilage samples were collected for macroscopic, histological and micro-CT evaluations.

Micro-CT

The microstructure of femurs of rat was examined by using the micro-CT scanner (mCT80, Scanco Medical AG). The scanner was set at a 114-µA current, a 70-kV voltage, and a resolution of 15.6 µm per pixel. The images of 3D reconstruction were performed and captured with Scanco Medical software.

Supplementary Figures

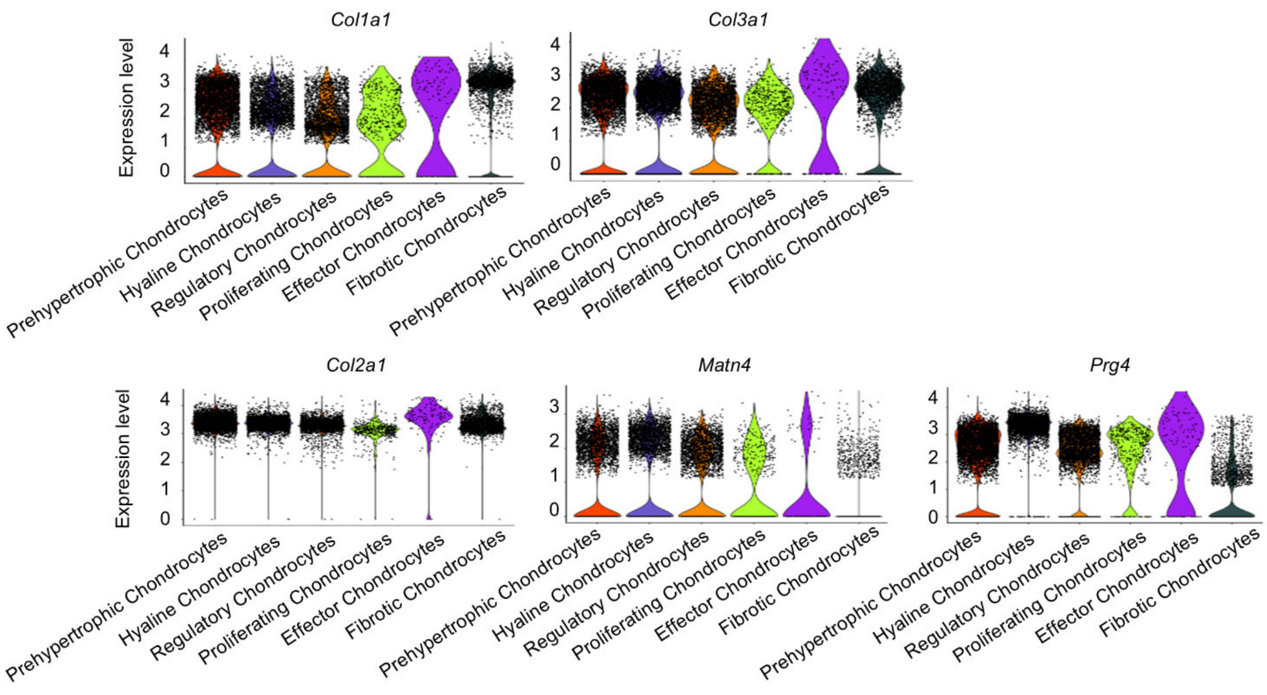

Figure S1.

Violin plots showing the gene expression levels of fibrotic chondrocyte markers (*Col1a1, Col3a1*) and hyaline chondrocyte markers (*Col2a1, Matn4, Prg4*) between different chondrocyte subclusters.

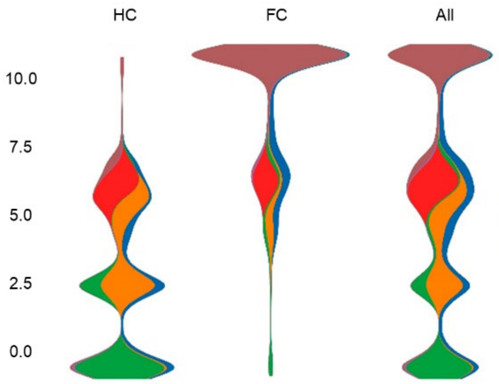

Figure S2.

Pseudo-time differentiation trajectory analysis of chondrocyte subclusters.

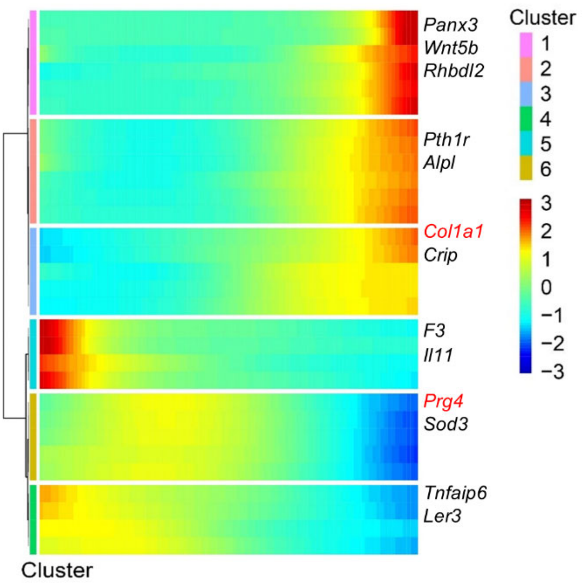

Figure S3.

The top genes expressed in different chondrocyte subclusters on a pseudo-time scale in HC and FC.

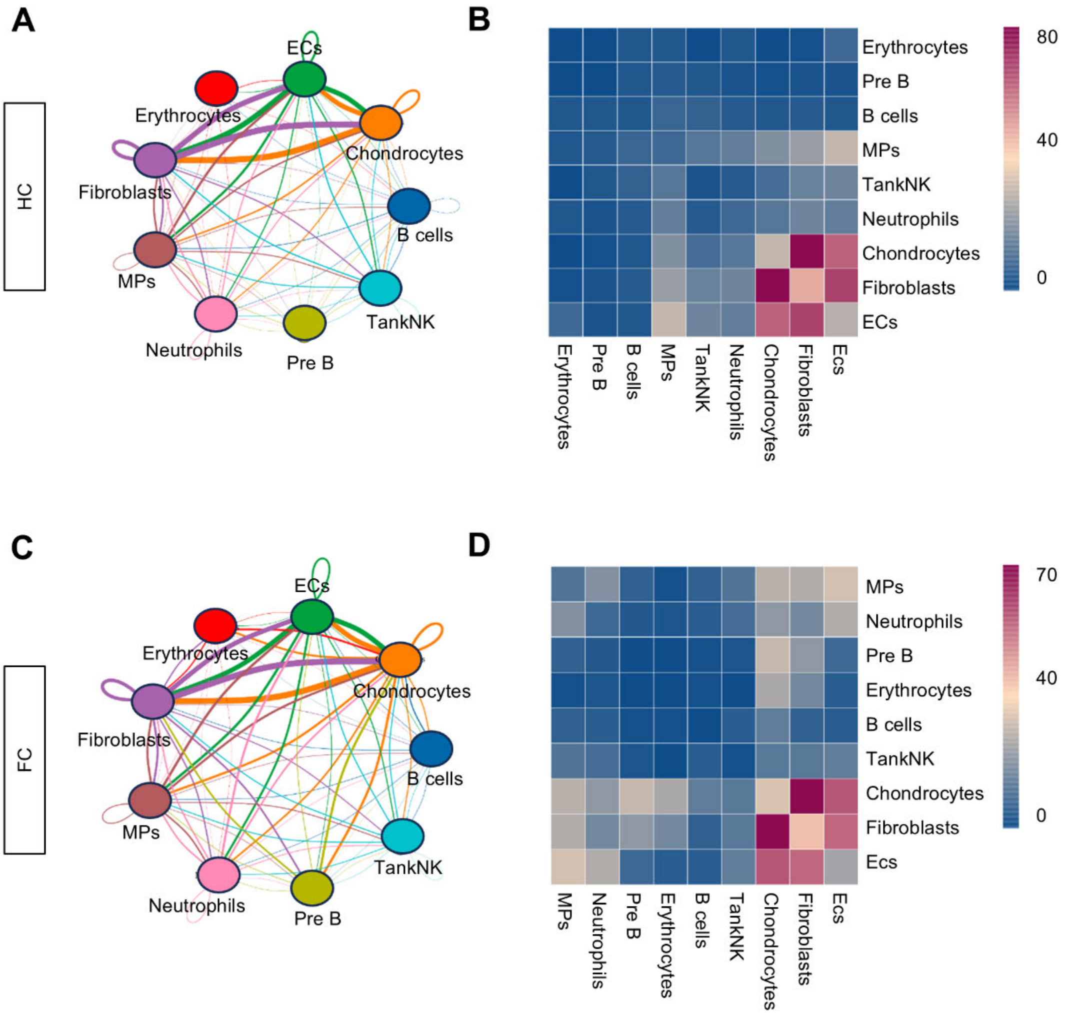

Figure S4.

(A-B) The cross network of different cell clusters in HC group. (C-D) The cross network of different cell clusters in FC group.

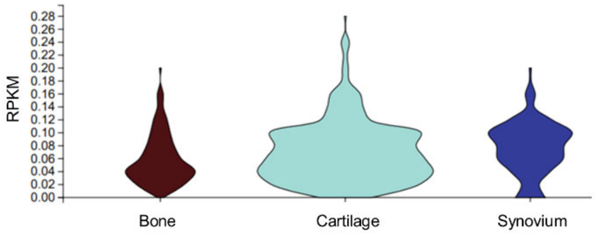

Figure S5.

The expression of *Kazald1* in human musculoskeletal tissues, generated using the MSdb database at https://www.msdb.org.cn/ [1].

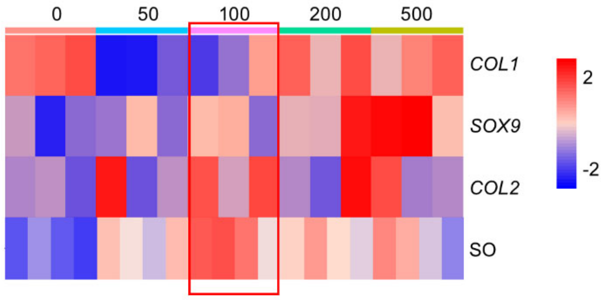

Figure S6.

Dose–effect heatmap analysis of Kazald1 treatment in human chondrocytes.

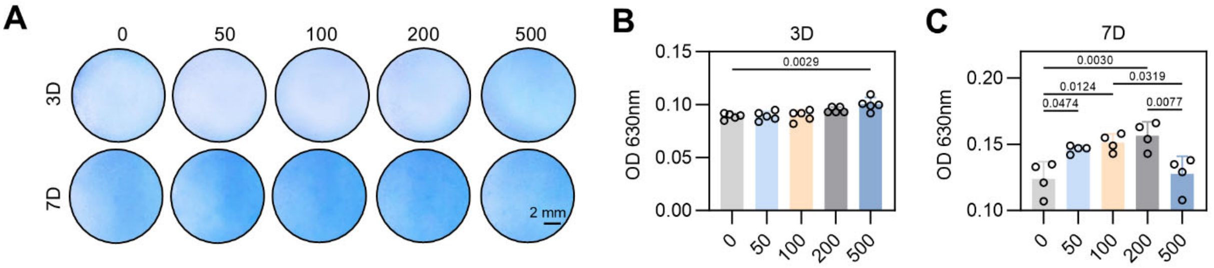

Figure S7.

(A) Alcian blue staining for human chondrocytes treated with different concentrations of Kazald1 protein, scale bar = 2 mm. (B-C) Quantification of Alcian blue staining on days 3 and 7 (n = 4/5 per group). Results are shown as mean ± SD.

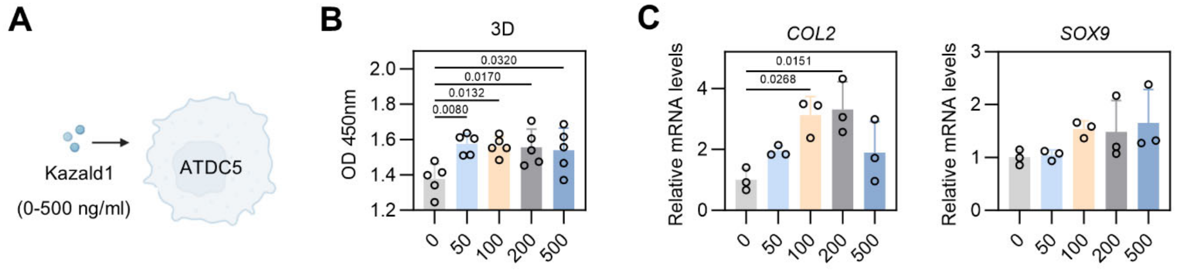

Figure S8.

(A) Schematic of *in vitro* cell experiments to assess the effect of Kazald1 on mouse ATDC5 chondrocytes. (B) CCK-8 assay evaluating the proliferation of mouse chondrocytes treated with different concentrations of Kazald1 protein on days 3 (n = 5 per group). (C) Gene expression levels of *COL2* and *SOX9* on day 3 (n = 3 per group). Results are shown as mean ± SD.

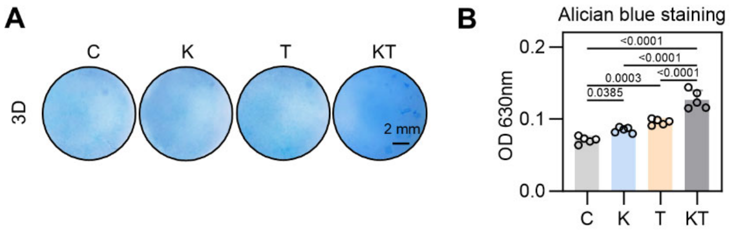

Figure S9.

(A-B) Alcian blue staining and quantification for human chondrocytes treated with Kazald1, TGF-β or Kazald1+ TGF-β on day 3, scale bar = 2 mm (n = 5 per group).

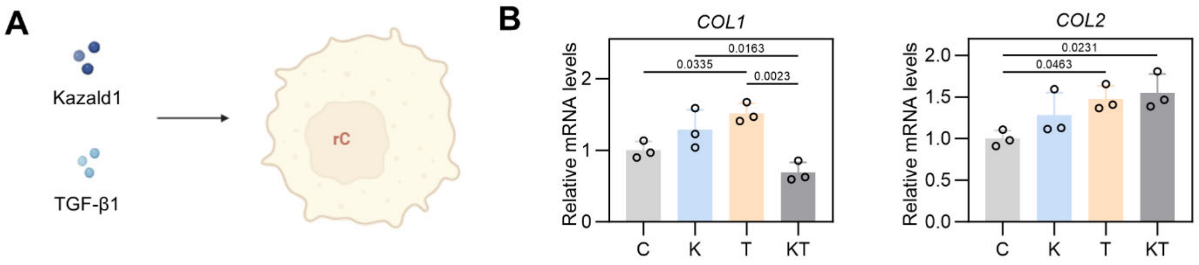

Figure S10.

(A) Schematic of *in vitro* cell experiments to assess the synergistic effect of Kazald1 and TGF-β1 on rat primary chondrocytes. (B) Gene expression levels of *COL1* and *COL2* on day 3 (n = 3 per group). Results are shown as mean ± SD.

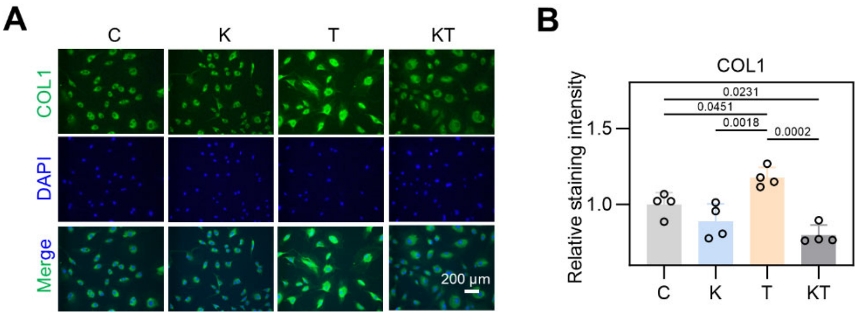

Figure S11.

(A) Immunofluorescent staining for COL1 in rat chondrocytes on day 3. (B) Quantification of COL1 immunofluorescent staining (n = 4 per group), scale bar = 200 μm. Results are shown as mean ± SD.

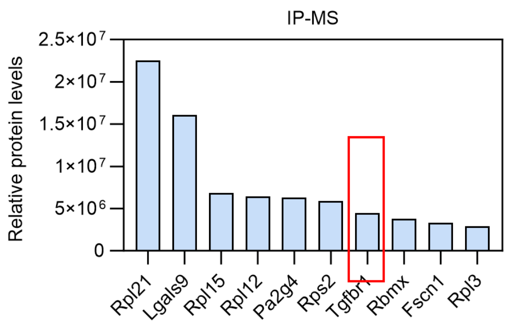

Figure S12.

Representative IP-MS spectrum showing the top ten proteins with the highest binding affinity to Kazald1.

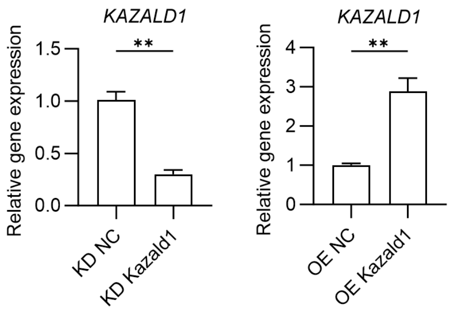

Figure S13.

Gene expression of *KAZALD1* in human chondrocytes treated with shKazald1 or AAV-shKazald1 virus (n = 3 per group). Results are shown as mean ± SD.

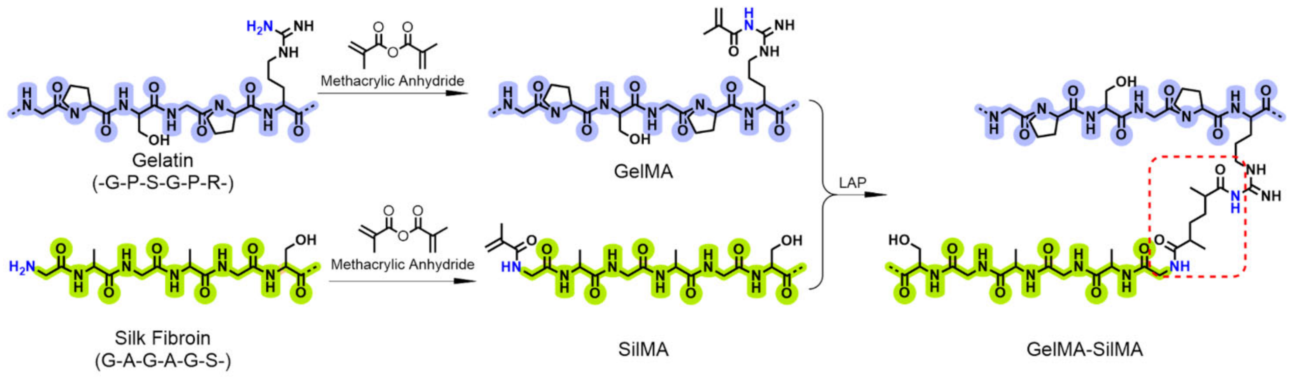

Figure S14.

Schematic of the fabrication process for the GelMA-SilMA composite hydrogel.

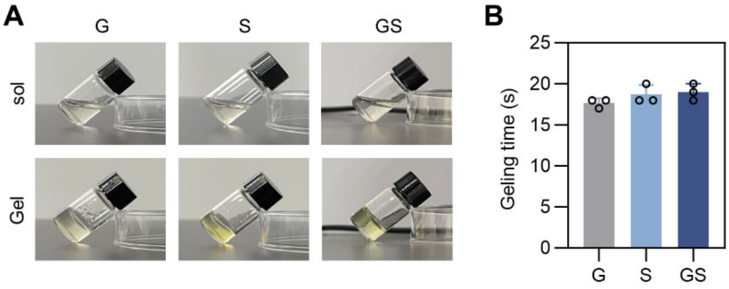

Figure S15.

(A) Sol-gel transition of GelMA, SilMA and GelMA-SilMA hydrogels. (B) Gelation time of different hydrogels (n = 3 per group).

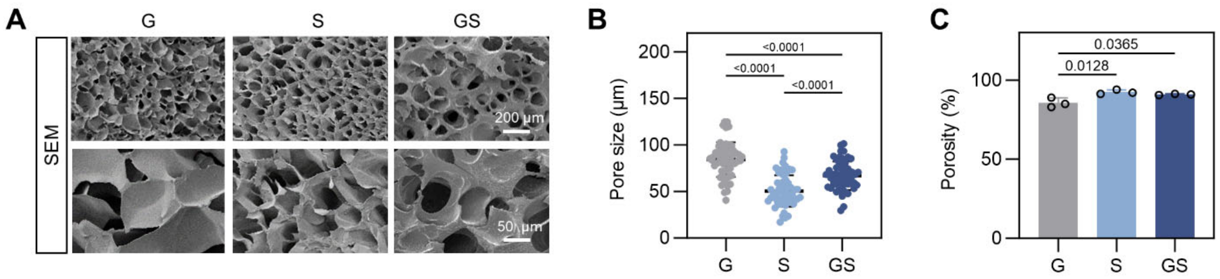

Figure S16.

(A) Microstructure of lyophilized GelMA, SilMA and GelMA-SilMA hydrogels from SEM images, scale bar = 200 μm. (B) The pore size of GelMA, SilMA and GelMA-SilMA hydrogels quantified from SEM images with Image J (n = 3 per group, 50 pores per sample). (C) The porosity of GelMA, SilMA and GelMA-SilMA hydrogels (n = 3 per group). Results are shown as mean ± SD.

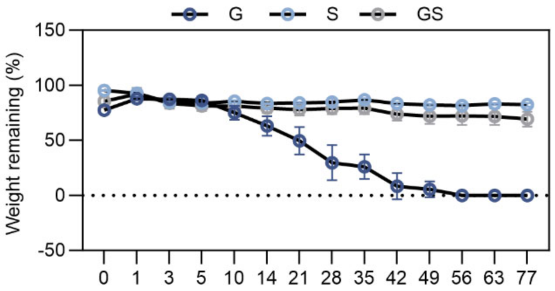

Figure S17.

In vitro degradation curves of GelMA, SilMA and GelMA-SilMA hydrogels in PBS at 37 °C (n = 3 per group). Results are shown as mean ± SD.

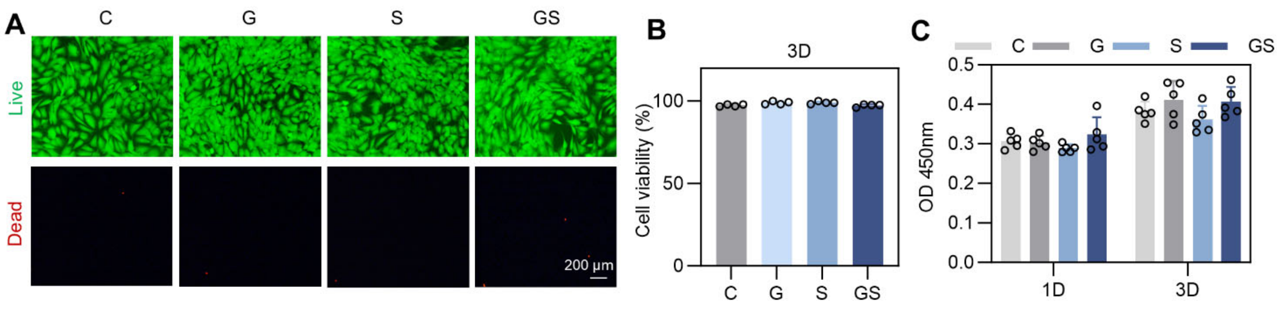

Figure S18.

(A) Live/dead staining evaluating the cell viability of human chondrocytes cultured in extracts of GelMA, SilMA and GelMA-SilMA hydrogels on day 3, scale bar = 200 μm. (B) Cell viability of human chondrocytes quantified from Live/dead staining (n = 4 per group). (C) CCK-8 assay evaluating the proliferation of human chondrocytes cultured in extracts of GelMA, SilMA and GelMA-SilMA hydrogels on day 1 and 3 (n = 5 per group). Results are shown as mean ± SD.

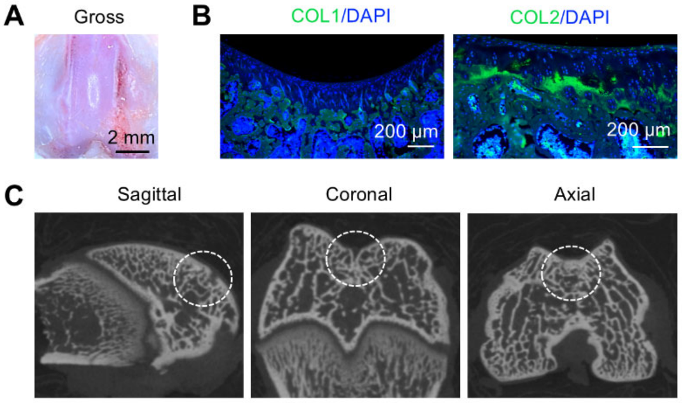

Figure S19.

(A) Macroscopic views of repaired cartilage treated with Gel@KT hydrogels after 12 weeks. (B) Immunofluorescent staining for COL1 and COL2 of repaired cartilage in Gel@KT groups, scale bar = 200 μm. (C) Micro-CT images of sagittal, coronal, and axial planes.

Table S1. List of primers for qPCR analysis.

Gene	Species	Forward	Reverse
GAPDH	Mouse	GCAAGTTCAACGGCACAG	CGCCAGTAGACTCCACGAC
COL2	Mouse	CCTCCGTCTACTGTCCACTGA	ATTGGAGCCCTGGATGAGCA
SOX9	Mouse	CGGCTCCAGCAAGAACAAG	GCGCCCACACCATGAAG
GAPDH	Rat	GCAAGTTCAACGGCACAG	CGCCAGTAGACTCCACGAC
COL2	Rat	ACGGCAACCTGGCTCCCAACA C	GCGTGAGGTCTTCTGTGATCG
COL1	Rat	TGGATGGCTGCACGAGT	TTGGGATGGAGGGAGTTTA
KAZALD1	Human	TGGGCCATGCTATTCTCAGAC	AGCCAGTCACCTCAAACCTC

Table S2. Antibodies used for IF, WB, IP and IHC staining.

Antibody	Company	Code
COL2	Proteintech	28459-1-AP
ACAN	Proteintech	13880-1-AP
COL1	Proteintech	14695-1-AP
Akt	Proteintech	10176-2-AP
p-Akt	Proteintech	28731-1-AP
Smad3	ABcolnal	A19115
p-Smad3	ABcolnal	AP0727
β-actin	Proteintech	20536-1-AP
PRG4	Affinity	DF13331
TGFβ receptor I	Abcam	ab235578
CoraLite488-conjugated Goat Anti-Rabbit IgG (H + L)	Proteintech	SA00013-2
HRP-labeled Goat Anti-Mouse IgG (H+L)	Beyotime	A0354
HRP-labeled Goat Anti-Rabbit IgG (H+L)	Beyotime	A0352
HRP-conjugated Mouse Anti-Rabbit IgG Light Chain	Abclonal	AS061

Table S3. ICRS macroscopic evaluation of cartilage repair.

Cartilage repair assessment ICRS	Points
Degree of defect repair	
In level with surrounding cartilage	4
75% repair of defect depth	3
50% repair of defect depth	2
25% repair of defect depth	1
0% repair of defect depth	0
Integration to border zone	
Complete integration with surrounding cartilage	4
Demarcating border < 1 mm	3
3/4th of graft integrated, 1/4th with a notable border > 1 mm width	2
1/2 of graft integrated with surrounding cartilage, 1/2 with a notable border > 1 mm	1
From no contact to 1/4th of graft integrated with surrounding cartilage	0
Macroscopic appearance	
Intact smooth surface	4
Fibrillated surface	3
Small, scattered fissures or cracs	2
Several, small or few but large fissures	1
Total degeneration of grafted area	0
Overall repair assessment	
Grade I: normal	12
Grade II: nearly normal	11–8
Grade III: abnormal	7–4
Grade IV: severely abnormal	3–1

Table S4. The information of patients.

	Sex	Age	Kellgren-Lawrence grade	BMI	Source
1.	Male	70	III	24.13	Nanjing Drum Tower Hospital
2.	Female	68	III	24.46	Nanjing Drum Tower Hospital
3.	Female	68	III	23.59	Zhongda Hospital Affiliated to Southeast University
4.	Male	59	III	26.4	Zhongda Hospital Affiliated to Southeast University
5.	Female	62	IV	32.05	Zhongda Hospital Affiliated to Southeast University
